# Supplementary material for: Parallel CRISPR screens reveal pathways controlling the cell surface levels of the attractant receptor FPR1
Source: Commun Biol. 2026 Mar 25;9:668. doi: 10.1038/s42003-026-09878-3 (PMC13181108; doi:10.1038/s42003-026-09878-3)
Supplement: Supplementary file 1 — Supplemental Material [file 42003_2026_9878_MOESM1_ESM.pdf]

Parallel CRISPR screens reveal pathways controlling the cell surface levels of the attractant receptor FPR1

Emel Akdoğan[1,*], Stefan M. Lundgren[1], Roarke A. Kamber[2,3], Michael C. Bassik[2], Sean R. Collins[1,*]

[1] Department of Microbiology and Molecular Genetics, University of California, Davis, Davis, CA, USA
[2] Department of Genetics, Stanford University, Stanford, CA, USA
[3] Current address: Department of Anatomy, University of California San Francisco, San Francisco, CA, 94143, USA
[*] Corresponding authors: srcollins@ucdavis.edu (Lead contact), emelakdogann@gmail.com

Supplementary Materials

List of Supplementary Data associated with this paper:

Supplementary Data 1. Screen scores for all sgRNAs.
Supplementary Data 2. Screen scores for all genes.
Supplementary Data 3. Negative hits identified in the basal surface FPR1 expression screen.
Supplementary Data 4. Positive hits identified in the basal surface FPR1 expression screen.
Supplementary Data 5. Negative hits identified in the post-stimulation surface FPR1 expression screen.
Supplementary Data 6. Positive hits identified in the post-stimulation surface FPR1 expression screen.
Supplementary Data 7. Negative hits identified by the integrated analysis of two screens for FPR1 internalization.
Supplementary Data 8. Positive hits identified by the integrated analysis of two screens for FPR1 internalization.
Supplementary Data 9. Numerical source data.

Supplementary Table 1. Primer and sgRNA target sequences used in this study. Sequences given in 5' to 3' direction. Targeting sequences are listed for CRISPR and CRISPRi sgRNAs.

Primer or Guide Name	Sequence (5' -> 3')
oEA20 (3XHA-FPR1 Fwd)	cttccatttcaggtgtcgtgagGATCCATGTACCCATACGATG
oEA28 (3XHA-FPR1 Rvs)	ccgtcgactctagagcggccgcTCACTTTGCCTGTAACTCCACCTctg
GFP CRISPR guide	ggcgagggcgatgccaccta
ARRB1 CRISPR guide #1	GGGGCTTCTTGTCCTCGG

ARRB1 CRISPR guide #2	GTCCCGCTTTCCCAGGTAGA
ARRB2 CRISPR guide #1	GACGGAGCATGGAAGATTCT
ARRB2 CRISPR guide #2	GGACCGGCTGCTGAGGAAGC
Safe-targeting (ST) guide	GAGCAGAGACCTCCTGAACC
ARRB1 Amplicon Seq Fwd	ACACTCTTTCCCTACACGACGCTCTTCCGATCTCCCACAGAGCTT GGGACAG
ARRB1 Amplicon Seq Rvs	GACTGGAGTTCAGACGTGTGCTCTTCCGATCTCCTCACCGCCCTC TTATG
ARRB2 Amplicon Seq Fwd	ACACTCTTTCCCTACACGACGCTCTTCCGATCTGCGTCTCCAGCC TCTTAG
ARRB2 Amplicon Seq Rvs	GACTGGAGTTCAGACGTGTGCTCTTCCGATCTCATGTCCTGCCCT AGGAG
ADRBK1 (GRK2) CRISPRi guide #1	GgCCCCGACTGCAGTCCCGG
ADRBK1 (GRK2) CRISPRi guide #2	GCCCGCCGGGACTGCAGTCG
ADRBK2 (GRK3) CRISPRi guide #1	GTACGGTCGCCCTCCCCTCG
ADRBK2 (GRK3) CRISPRi guide #2	GCCCTCGTGGCCACCCCGAG
GRK5 CRISPRi guide #1	Ggagtgacagagacacgcgg
GRK5 CRISPRi guide #2	Gggggagggggacacaga
GRK6 CRISPRi guide #1	GCGAtcggcgcggctcggcg
GRK6 CRISPRi guide #2	GgctcgCAGTGAccgcgccg
Non-targeting (NT) guide	GTGCACCCGGCTAGGACCGG
oEA127 (GRK2 qPCR Fwd)	ATGGAGAAGAGCAAGGCCAC
oEA128 (GRK2 qPCR Rvs)	ATGACACTGCGGATGCTGG
oEA129 (GRK6 qPCR Fwd)	ACCGACCAGGACTTCTACCA
oEA130 (GRK6 qPCR Rvs)	CGGTCTCCACCATCTCGTTC
oEA290 (GRK3 qPCR Fwd)	TGACCGAATGACACTCACCG
oEA291 (GRK3 qPCR Rvs)	TTACTTCCTGTGAGCCGCC
oEA292 (GRK5 qPCR Fwd)	CTGGACTCCGTGGCAGAATA
oEA293 (GRK5 qPCR Rvs)	TGGCCAACTTGGGCTATGAA
G6PD qPCR Fwd	gtgacctggccaagaagaag
G6PD qPCR Rvs	gaagggctcactctgtttgc
oSRC1 (Sequencing Primer)	GCCACTTTTTCAAGTTGATAACGGACTAGCCTTATTTAAACTTGCTA TGCTGTTTCCAGCTTAGCTCTTAAAC
oSRC2 (PCR 1 Forward)	aggcttggatttctataacttcgtatagcatacattatac
oSRC3 (PCR 1 Reverse)	acatgcatggcggtaatacggttatc
oSRC4 (PCR 2 Reverse)	caagcagaagacggcatacgagatgcacaaaggaaactcaccct
oSRC16 (TruSeq Index 26)	aatgatacggcgaccaccgagatctacacGATCGGAAGAGCACACGTCTGAAC TCCAGTCACatgagcCGACTCGGTGCCACTTTTTC
oSRC17 (TruSeq Index 27)	aatgatacggcgaccaccgagatctacacGATCGGAAGAGCACACGTCTGAAC TCCAGTCACattcctCGACTCGGTGCCACTTTTTC
oSRC18 (TruSeq Index 28)	aatgatacggcgaccaccgagatctacacGATCGGAAGAGCACACGTCTGAAC TCCAGTCACcaaaagCGACTCGGTGCCACTTTTTC

oSRC19 (TruSeq Index 29)	aatgatacggcgaccaccgagatctacacGATCGGAAGAGCACACG TCTGAACTCCAGTCACcaactaCGACTCGGTGCCACTTTTTC
ARF1 CRISPR guide PAM	GCTGTCCACCACGAAGATC
ARF6 CRISPR guide PAM #1	GTTCAACGTATGGGATGT
ARF6 CRISPR guide PAM #2	GTTCAACGTATGGGATGT
ARF6 g1 Amplicon Seq Fwd	ACACTCTTTCCCTACACGACGCTCTTCCGATCTCAATGACCGGGA GATGAGG
ARF6 g1 Amplicon Seq Rvs	GACTGGAGTTCAGACGTGTGCTCTTCCGATCCTCTCCTTCCAGGG GATG
ARF6 g2 Amplicon Seq Fwd	ACACTCTTTCCCTACACGACGCTCTTCCGATCTTTGAAGCTGGGC CAGTCG
ARF6 g2 Amplicon Seq Rvs	GACTGGAGTTCAGACGTGTGCTCTTCCGATCTGCGCAGTCCACTA CGAAG
oEA310 (ARF6 qPCR Fwd)	GGATCCTCATGTTGGGCCTG
oEA311 (ARF6 qPCR Rvs)	CGTCTCCACGTTGAAACCCA
CSNK1A1 CRISPRi guide #1	GGGTCCCTCTGGGCCGGAGC
CSNK1A1 CRISPRi guide #2	GCGTGAAGAAGCGGCTCCCG
ZBTB7A CRISPRi guide #1	GccggcgcggcccccggcaC
ZBTB7A CRISPRi guide #2	GCCGCGCGAGGGAGCGACCA
MLST8 CRISPRi guide #1	GCGGAGCCGCCCGTAAGGTA
MLST8 CRISPRi guide #2	GCACTCCGCCGTGTCCAGGA
CCDC22 CRISPRi guide #1	GTCCCGGATGTGGGCTTCGG
CCDC22 CRISPRi guide #2	GCTCCGACACGGCTCCACCA
COMMD5 CRISPRi guide #1	GggacgccacCCGACAGGAG
COMMD5 CRISPRi guide #2	GAGGCGCAGACCGTCCACAC
PTEN CRISPRi guide #1	GCATGGCTGCAGCTTCCGAG
PTEN CRISPRi guide #2	GCGAGGGAGATGAGAGACGG
GALE CRISPRi guide #1	GGTGCCTCTGCAGCAAGCGT
GALE CRISPRi guide #2	GCGGGAACCCGAGCAGGTTC
SLC35A2 CRISPRi guide #1	GTCTGCCCGAGCAGTCGCAT
SLC35A2 CRISPRi guide #2	GGTCGGGATCGCTGGGAGTC
SIK3 CRISPRi guide #1	GTCACTGCACAACAAGATGG
SIK3 CRISPRi guide #2	GTTGTGCAGTGAAACCTCCG
RAB11A CRISPRi guide #1	GGCGCGGCCGAGGAGCGAAA
RAB11A CRISPRi guide #2	GCGGCCGAGGAGCGAAAGGG
EXOC2 CRISPRi guide #1	GGGCGGAAGTGAGGTGCCGG
EXOC2 CRISPRi guide #2	GCTGGCGGCCGCGGAGTCTT
RGL2 CRISPRi guide #1	GCGGAGACCGACGGCAACAG
RGL2 CRISPRi guide #2	GGTCCGGGGCCGGAGACCGA

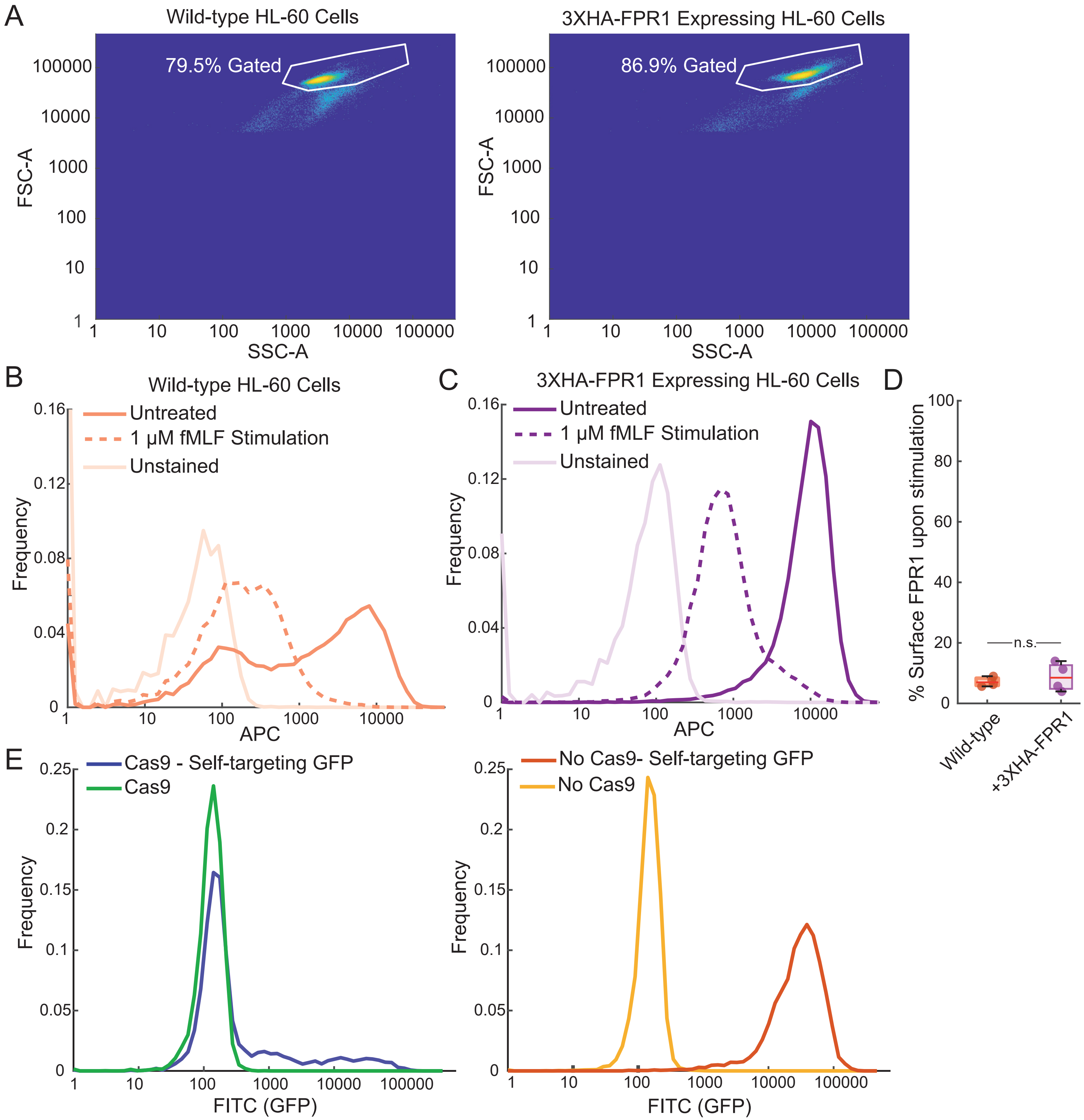

Supplementary Figure 1. Optimization of tools used in this study. (A) Example gating strategy for flow cytometry measurements. Comparison of FPR1 internalization in (B) wild-type (WT) and (C) 3XHA-FPR1 expressing differentiated HL-60 cells. Cells were untreated or stimulated with 100 nM fMLF and stained for remaining surface FPR1. Note that basal surface expression of FPR1 is much more uniform in cells with the 3XHA-FPR1 construct. (D) FPR1 endocytosis capacities of WT and 3XHA-FPR1 expressing differentiated HL-60s were compared as described in B and C (n=4). Data for wild-type is a subset of the dataset used in Figure 1, and the data is reused in this figure for comparison purposes. Throughout this study, we use Mann-Whitney U test for pairwise comparison of the two samples indicated in figures (n.s. $p>0.05$, *$p<0.05$, **$p<0.01$, ***$p<0.001$), and dots represent different biological replicates. The central line indicates the median in the box plots. False discovery rate was controlled within each dataset by the Benjamini-Hochberg procedure ($q<0.02$ for all cases where $p<0.01$). (E) Representation of knockout efficiency using a self-targeting GFP construct that expresses GFP and an sgRNA that targets GFP expression. >80% GFP signal is lost when this construct is expressed along with Cas9.

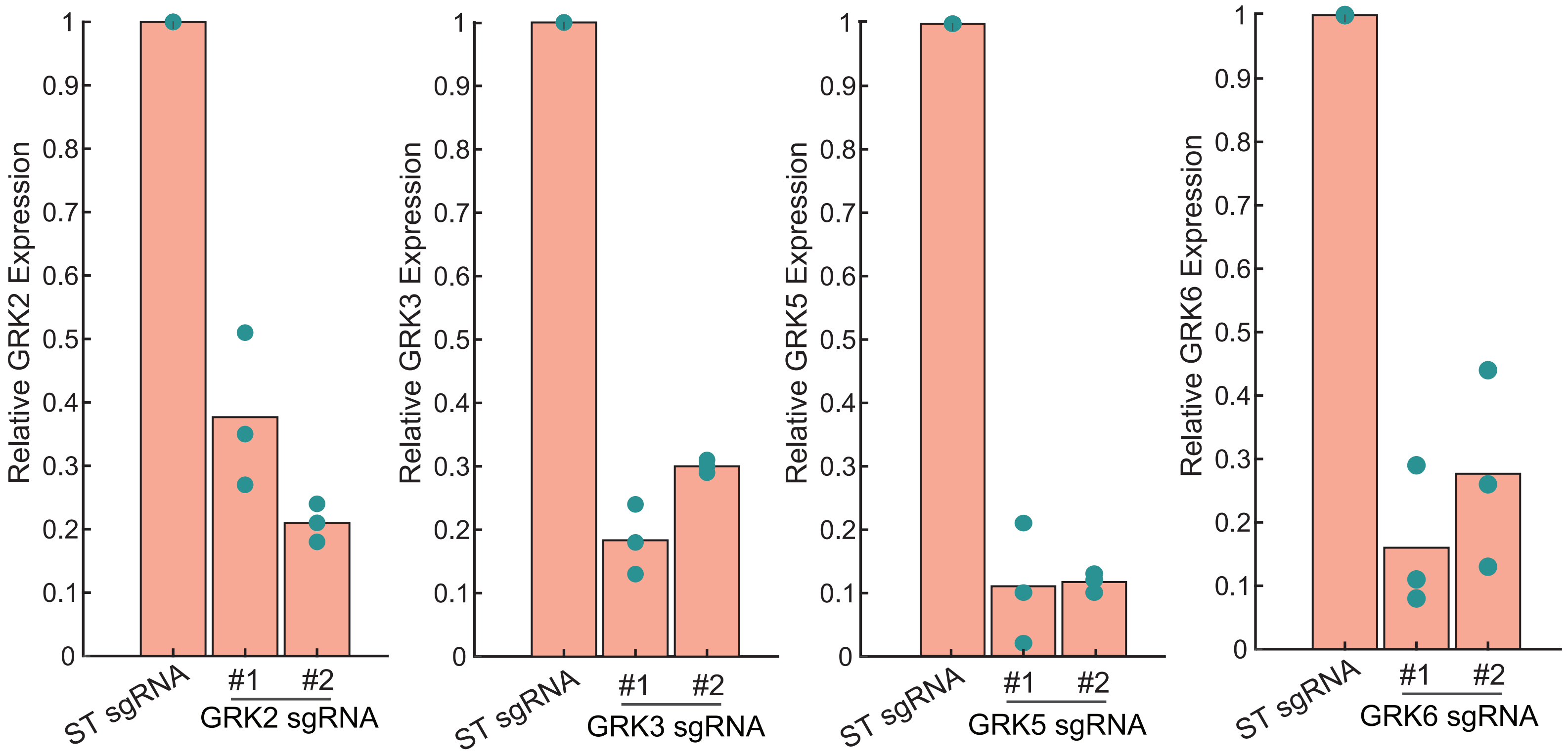

Supplementary Figure 2. Knockdown efficiencies in dHL-60 cells expressing dCas9 and GRK sgRNAs assessed by RT-qPCR. Dots represent three biological replicates for each condition. Three technical replicates are done in all experiments, and mean Cp values were used for subsequent calculations.

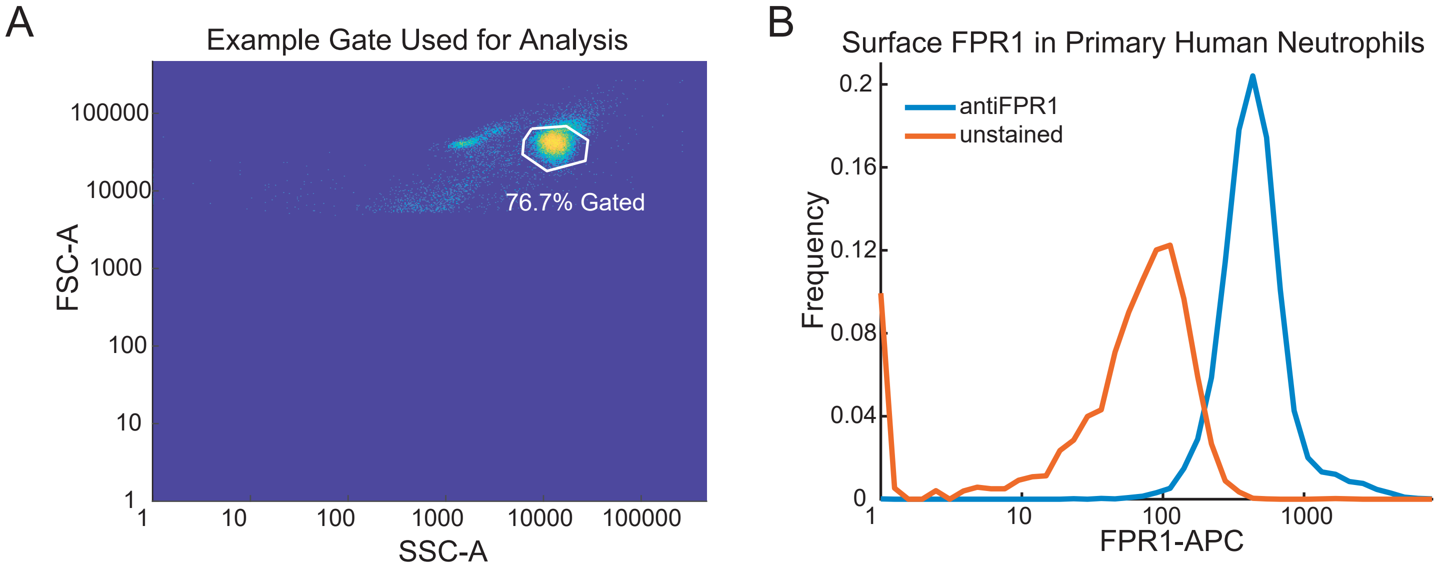

Supplementary Figure 3. Purity and uniform FPR1 staining of isolated human neutrophils. (A) Gating strategy used for freshly isolated human neutrophils in flow cytometry experiments. (B) Uniform surface FPR1 staining of isolated neutrophils. Details of the protocol for neutrophil isolation are given in Methods.

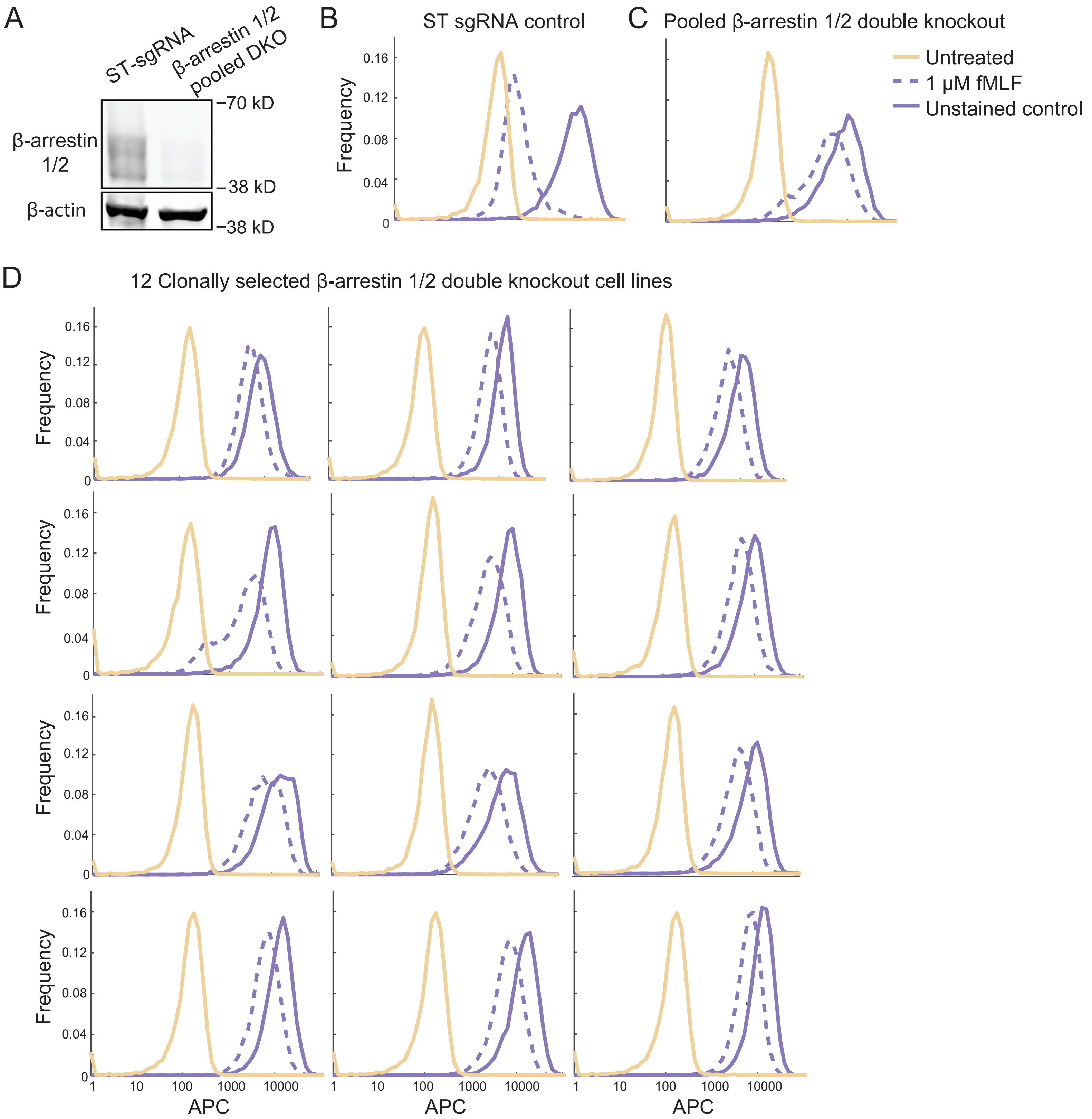

Supplementary Figure 4. Pooled β-arrestin DKO is efficient and has a similar endocytosis phenotype to individual clones. (A) Western blotting to confirm β-arrestin 1/2 double knockout efficiency. Cell lysates were used to assess β-arrestin levels. Staining for loading control (β-actin) was done on the same membrane. Image is representative of n=3 biological replicates. Clonally selected β-arrestin double CRISPR knockout cells are compared to pooled double β-arrestin knockout cell lines using the FPR1 endocytosis assay. Cells were untreated or stimulated with 1 µM fMLF. (B) A representative plot showing fMLF-induced decrease in surface FPR1 in the control sgRNA expressing cells. (C) Pooled double β-arrestin knockout cells have a smaller shift after fMLF stimulation. (D) Representative plots obtained from twelve clonally selected β-arrestin double knockout cell lines. Phenotypes observed in clonally selected and pooled knockout cell lines resemble each other.

A ARRB1 Reference Sequence GGCCCCCG-AGGACAAGAAGCCCCTGA

Cell Line	# of Reads	Sequence	Type of Mutation
ST sgRNA	15142	GGCCCCCG-AGGACAAGAAGCCCCTGA	*wild type*
1.42% mutant	14909	GGCCCCCG-AGGACAAGAAGCCCCTGA	*wild type*
ARRB1 sgRNA Pool KO 97.56% mutant	4433	GG-------------------CCCCTGA	*deletion*
	4284	GG-------------------CCCCTGA	*deletion*
	1745	GGCCCCCGAAGGACAAGAAGCCCCTGA	insertion, *frameshift*
	1651	GGC------------------CCCCTGA	*deletion, frameshift*
ARRB1 sgRNA Clone #D4 99.66% mutant	22557	GGCCCCCGAAGGACAAGAAGCCCCTGA	insertion, *frameshift*
ARRB1 sgRNA Clone #E3 99.88% mutant	17611	GG-------------------CCCCTGA	*deletion*
	14327	GGCCCCCG--GGACAAGAAGCCCCTGA	*deletion, frameshift*
	3080	GG-------------------CCCCTGA	
	2616	GGCCCCCG--GGACAAGAAGCCCCTGA	
ARRB1 sgRNA Clone #E7 99.90% mutant	15497	GGC------------------CCCCTGA	*deletion, frameshift*
	13688	GGCCCCCG--GGACAAGAAGCCCCTGA	*deletion, frameshift*
	6535	GGC------------------CCCCTGA	
	1416	GGCCCCCG--GGACAAGAAGCCCCTGA	

B ARRB2 Reference Sequence CCCAGAA-TCTTCCATGCTCCGTCAC

Cell Line	# of Reads	Sequence	Type of Mutation
ST sgRNA 1.20% mutant	59047	CCCAGAA-TCTTCCATGCTCCGTCAC	*wild type*
ARRB2 sgRNA Pool KO 89.46% mutant	13524	CCCAGAAATCTTCCATGCTCCGTCAC	insertion, *frameshift*
	11300	CCCAT-----------GCTCCGTCAC	*deletion*
	6449	CCCAGAA-TCTTCCATGCTCCGTCAC	*deletion, frameshift*
	5038	CCCAGA--TCTTCCATGCTCCGTCAC	deletion
ARRB2 sgRNA Clone #D4 99.94% mutant	45093	CCCAG------------CTCCGTCAC	*deletion, frameshift*
ARRB2 sgRNA Clone #E3 100% mutant	53656	CCCAT-----------GCTCCGTCAC	*deletion*
	1114	CCCAG------------CTCCGTCAC	
ARRB2 sgRNA Clone #E7 100% mutant	52551	CCCAGAAATCTTCCATGCTCCGTCAC	insertion, *frameshift*
	730	CCCAG------------CTCCGTCAC	

Supplementary Figure 5. Comparison of amplicon sequencing results for β-arrestin KO cell lines. Sequences with read numbers greater than 1% of the total reads obtained for the particular sample are presented for (A) β-arrestin1 and (B) β-arrestin2 in control sgRNA expressing cells, pooled and cloned β-arrestin DKO cell lines. Mutant percentage is calculated by subtracting the percentage of reads that contain the expected genomic sequence from 100. The targeted sequence by the sgRNA is highlighted in gray. Base-insertion (A, Adenine) is highlighted in green.

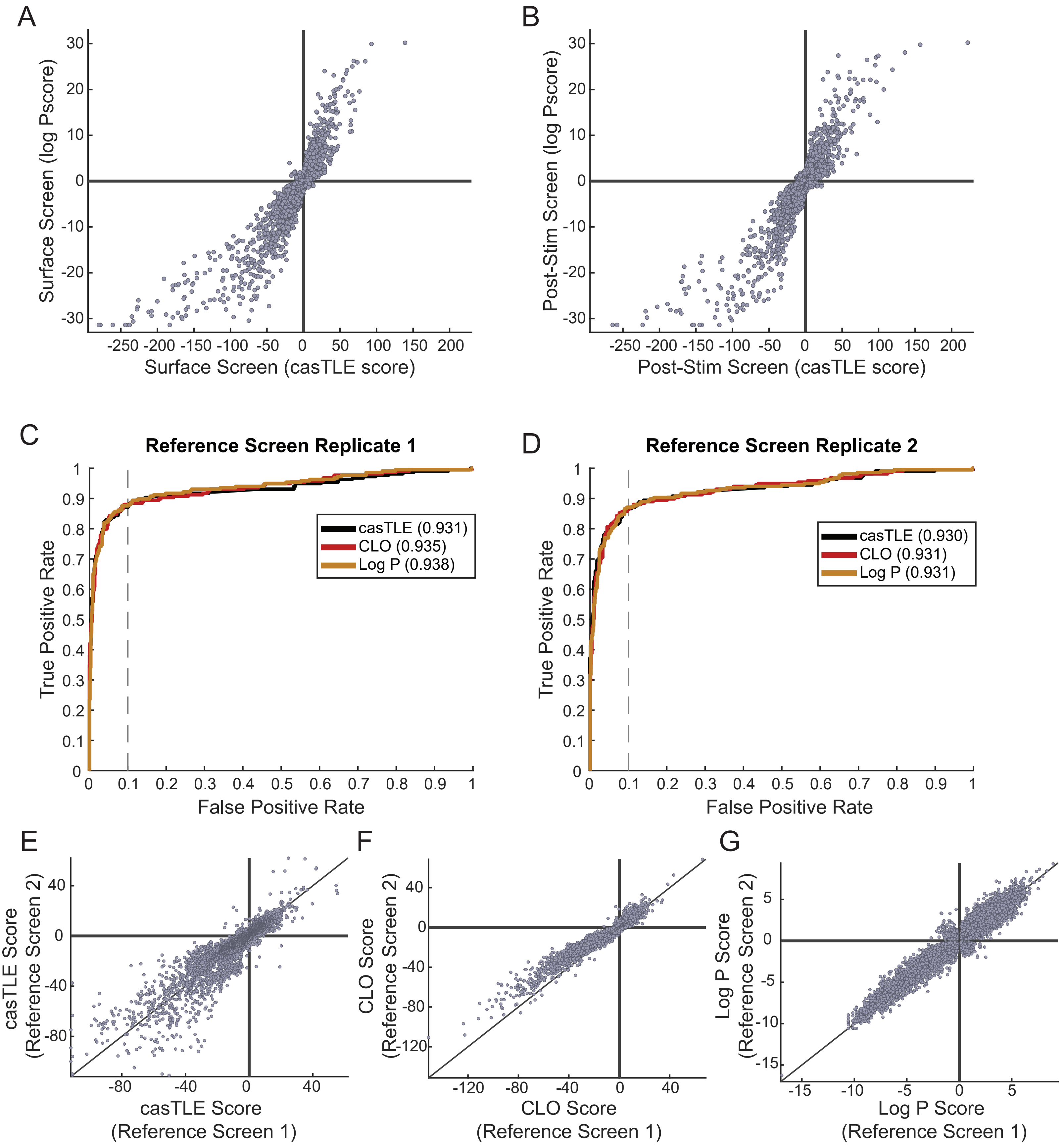

Supplementary Figure 6. Validation of the log-likelihood scoring system by comparison to the established casTLE analysis. Comparison of log P-scores and casTLE scores across all genes for single screens: (A) Surface Screen and (B) Post-stim Screen. Reanalysis of published gene essentiality screens[1] using our log likelihood scoring (log P, CLO) method and its comparison to casTLE analysis. ROC curves for identifying essential genes using these methods were calculated for screen replicate 1 (C) and replicate 2 (D). True and false positive rates were calculated as described previously[1]. Distribution and consistency of casTLE, log P and CLO scores across the two replicate gene essentiality screens[1] are shown in scatter plots for each scoring method (E-G).

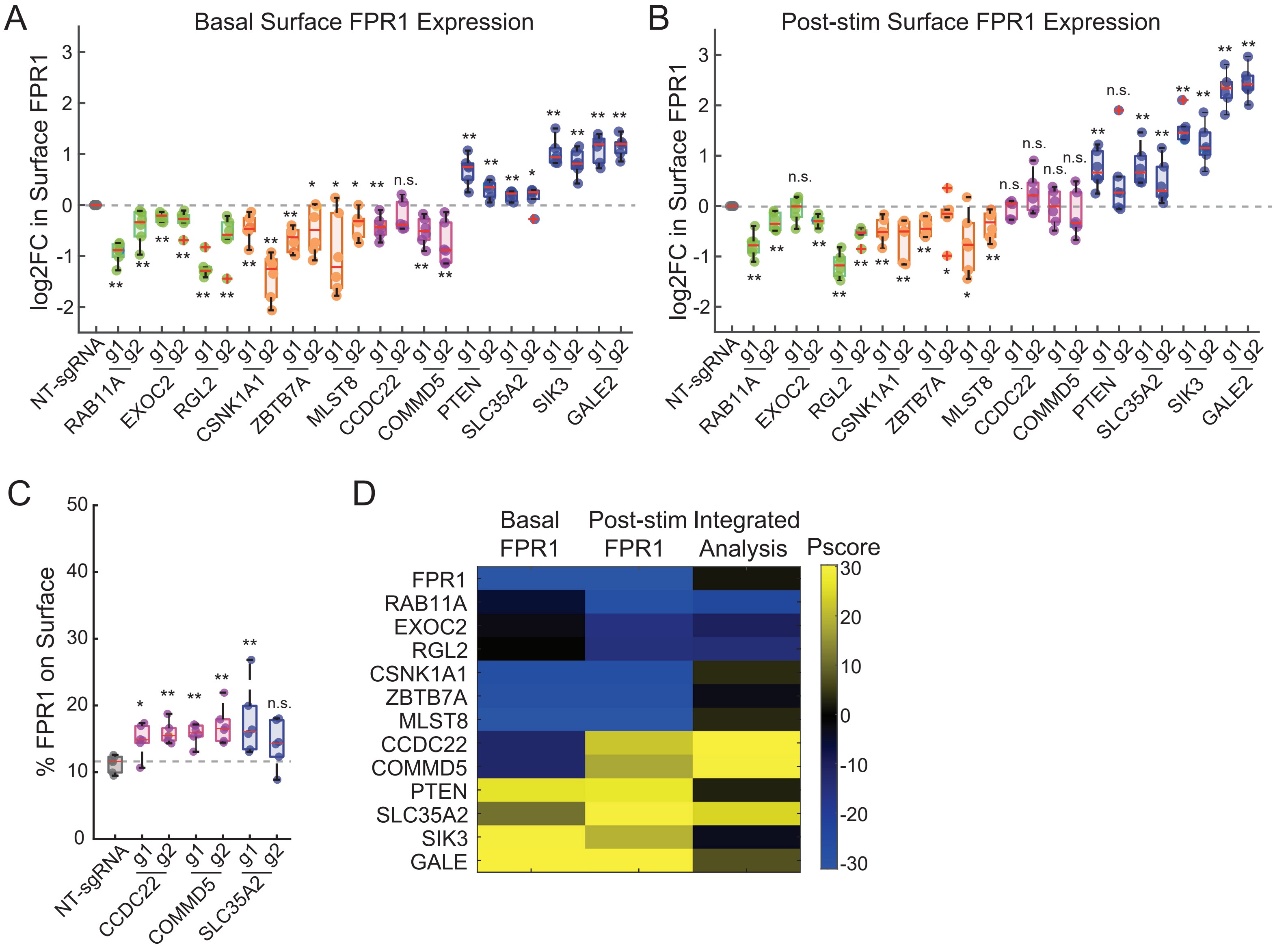

Supplementary Figure 7. Validation of top hits across different phenotypes from Surface FPR1 and Post-stim Surface FPR1 screens. Differences (log2 fold change, log2FC) in surface FPR1 levels are shown in (A) unstimulated and (B) stimulated (10 min, 100 nM fMLF) cells with CRISPRi knockdown of 12 genes (CSNK1A1, ZBTB7A, MLST8, CCDC22, COMMD5, PTEN, SLC35A2, SIK3, GALE, RAB11A, EXOC2, RGL2) with two independent guides per gene (indicated by g1 and g2). NT-sgRNA is a non-targeting control. (n=6 biological replicates, n.s. $p>0.05$, *$p<0.05$, **$p<0.01$) (C) Percentage of surface FPR1 remaining after stimulation are shown for genes identified as potential regulators of FPR1 internalization through integrated analysis of two screens. n=6 biological replicates. (D) A heatmap of log P-scores obtained from CRISPR screens is shown for the 12 genes tested. Blue represents a phenotype with low surface FPR1 and yellow represents high surface FPR1.

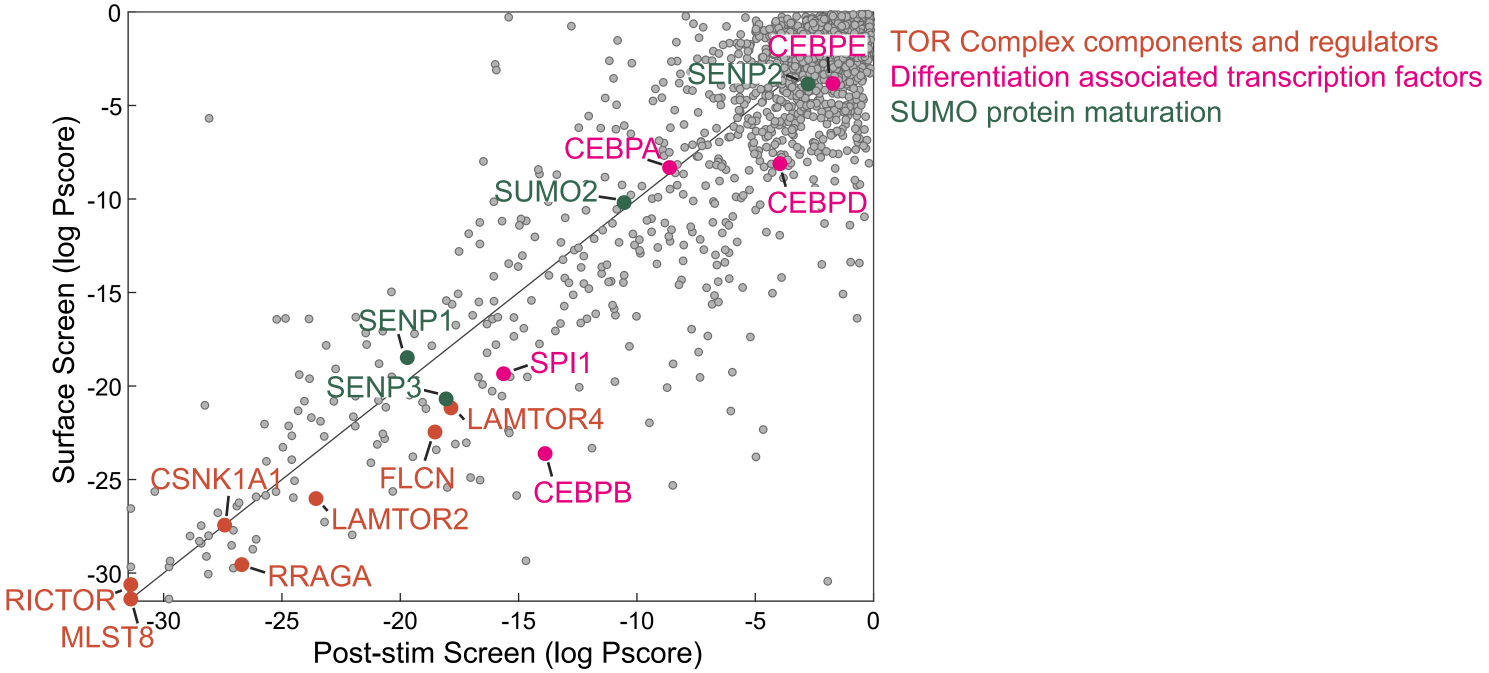

Supplementary Figure 8. Cell differentiation associated hits identified in the basal surface FPR1 screen. Plot showing the effect of the knockout of the TOR associated genes, neutrophil differentiation associated transcription factor encoding genes, and genes that function in the maturation of SUMO proteins on FPR1 surface levels in the basal (y-axis) and post-stimulation screens (x-axis).

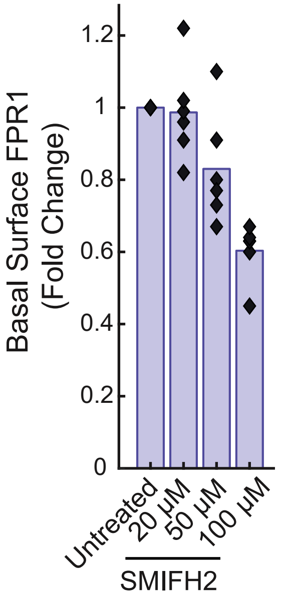

Supplementary Figure 9. High SMIFH2 concentrations cause decreases in basal surface FPR1. Fold change in surface FPR1 from the control is calculated for different concentrations of SMIFH2 treatment.

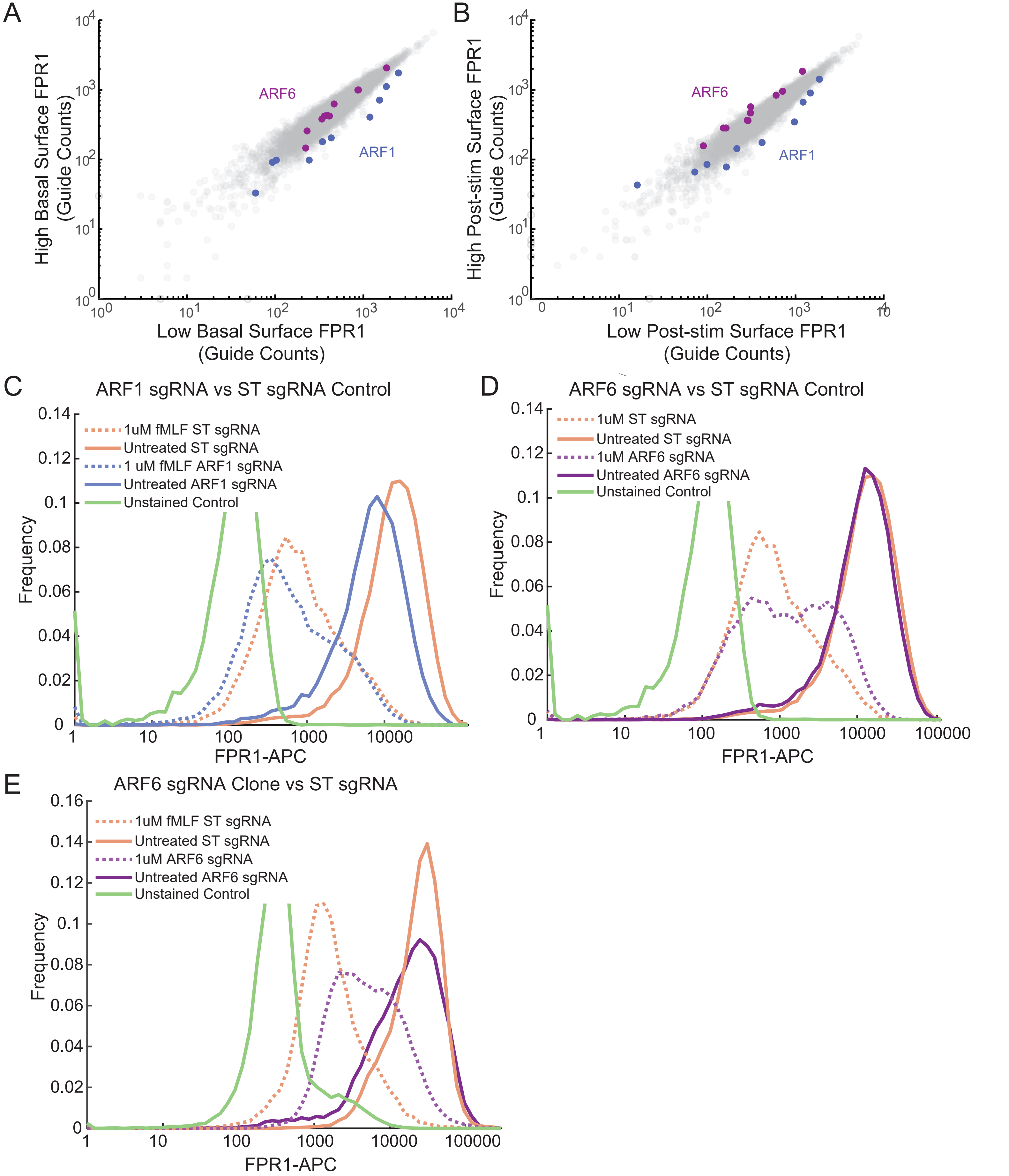

Supplementary Figure 10. Arf6 facilitates FPR1 internalization, while Arf1 takes roles in basal surface expression. Comparison of read counts for 10 unique sgRNAs targeting ARF1 or ARF6 to the guide counts of the control guides in (A) the basal surface FPR1 expression screen and (B) the post-stimulation FPR1 surface expression screen. Shift from the control guides indicates a lower or a higher surface FPR1 in the basal state or after stimulation. Comparison of FPR1 internalization in (C) pooled Arf1, (D) pooled Arf6 (sgRNA #2), and (E) clonally selected Arf6 knockout cells (sgRNA #2, Clone 9) versus ST-sgRNA expressing cells.

A

Reference Sequence CCCCACG-AGATCCAGGAGAAACTGGG

Cell Line	# of Reads	Sequence	Type of Mutation
ST sgRNA	23168	CCCCACA-AGATCCAGGAGAAACTGGG	*alt wt*
0.42% mutant	8153	CCCCACG-AGATCCAGGAGAAACTGGG	*wild type*
ARF6 sgRNA#1	18145	CCCCACA-AGATCCAGGAGAAACTGGG	*alt wt*
Pool KO	1547	CCCCACGAAGATCCAGGAGAAACTGGG	*insertion, frameshift*
95.15% mutant	1504	CCCCAC---GATCCAGGAGAAACTGGG	*deletion*
	1271	CC----------CCAGGAGAAACTGGG	*deletion, frameshift*
ARF6 sgRNA#1	16455	CCCCACA-AGATCCAGGAGAAACTGGG	*alt wt*
Clone #1	9209	CC----------CCAGGAGAAACTGGG	*deletion, frameshift*
96.04% mutant			
ARF6 sgRNA#1	19867	CCCCACA-AGATCCAGGAGAAACTGGG	*alt wt*
Clone #4	6836	CCCCACGAAGATCCAGGAGAAACTGGG	*insertion, frameshift*
92.68% mutant			

B

Reference Sequence TCAAGTTCAACGTATGGGAT-GTGGGC

Cell Line	# of Reads	Sequence	Type of Mutation
ST sgRNA	40494	TCAAGTTCAACGTATGGGAT-GTGGGC	*wild type*
0.28% mutant	40157	TCAAGTTCAACATATGGGAT-GTGGGT	*alt wt*
	1885	TCAAGTTCAACGTATGGGAT-GTGGGC	*wild type*
	1759	TCAAGTTCAACGTATGGGAT-GTGGGC	*wild type*
ARF6 sgRNA#2	19740	TCAAGTTCAACGTATGGGA--------	*deletion, frameshift*
Pool KO	8165	TCAAGTTCAACGTAT----T-GTGGGC	*deletion, frameshift*
99.20% mutant	6691	TCAAGTTCAACATGTG--------GGT	*deletion, frameshift*
	6127	TCAAGTTCAACATATG--------GGT	*deletion, frameshift*
ARF6 sgRNA#2	37695	---------------------------	*deletion, frameshift*
Clone #9	14408	TCAAGTTCAACATATGGGATTGTGGGT	*insertion, frameshift*
99.29% mutant	2524	TCAAGTTCAACATATG------TGGGT	*deletion*
	2090	TCAAGTTCAACATATG--------GGT	*deletion, frameshift*

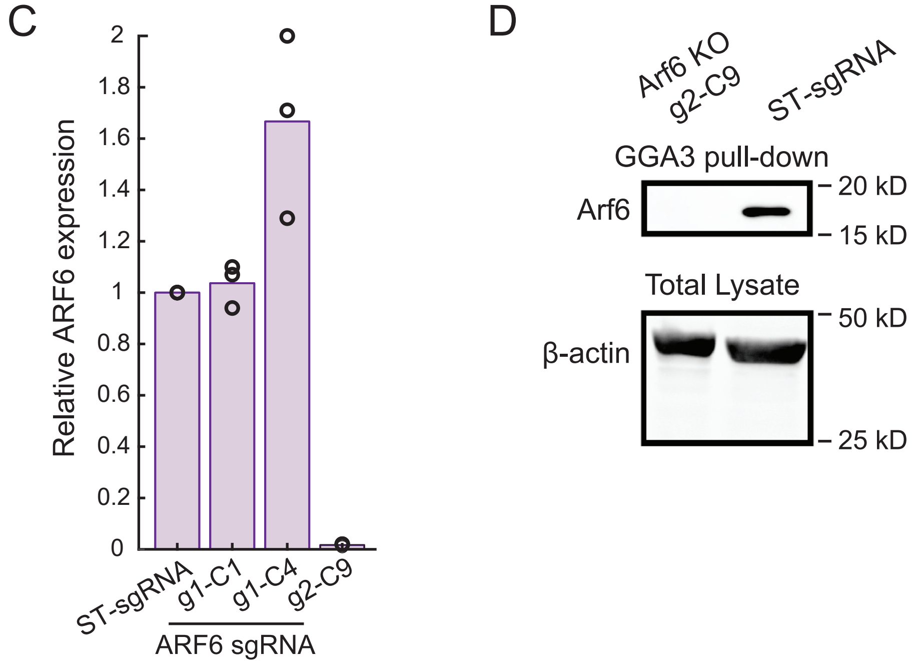

Supplementary Figure 11. Confirmation of Arf6 knockout efficiency. Sequences with read numbers greater than 1% of the total reads for each sample are presented. (A) ARF6 sequences for ST-sgRNA expressing cells, pooled ARF6 sgRNA#1, and Clones 1 and 4. (B) ARF6 sequences for ST-sgRNA expressing cells, pooled ARF6 sgRNA#2, and Clone 9. The targeted sequence by the sgRNA is highlighted in gray. We detected a frequent base variant in the control cell sequences from the reference sequence (G>A, highlighted in green), and we refer to this variant as the alternative wild-type (alt wt). Mutant percentage is calculated by subtracting the percentage of reads that contain the intact control sequence (wild-type or alt wt) from 100. (C) Western blotting to confirm Arf6 knockout efficiency at the protein level. We pulled down Arf GTPases in total cell lysates of ST-sgRNA expressing control cells and Arf6 knockout cells (Clone 9) using the Arf6 Pull-down Kit from Cytoskeleton Inc (see Methods for details). A sample from normalized total lysate was used to access total protein levels. All samples, pulldown and lysate, were run on the same gel and transferred to the same membrane, but they were probed with different antibodies (Arf6 antibody for pulldown samples and β-actin antibody for total lysate controls). Image is representative of n=3 biological replicates. (D) qPCR to detect presence of ARF6 mRNA in control sgRNA expressing and clonally selected ARF6 sgRNA expressing cells.

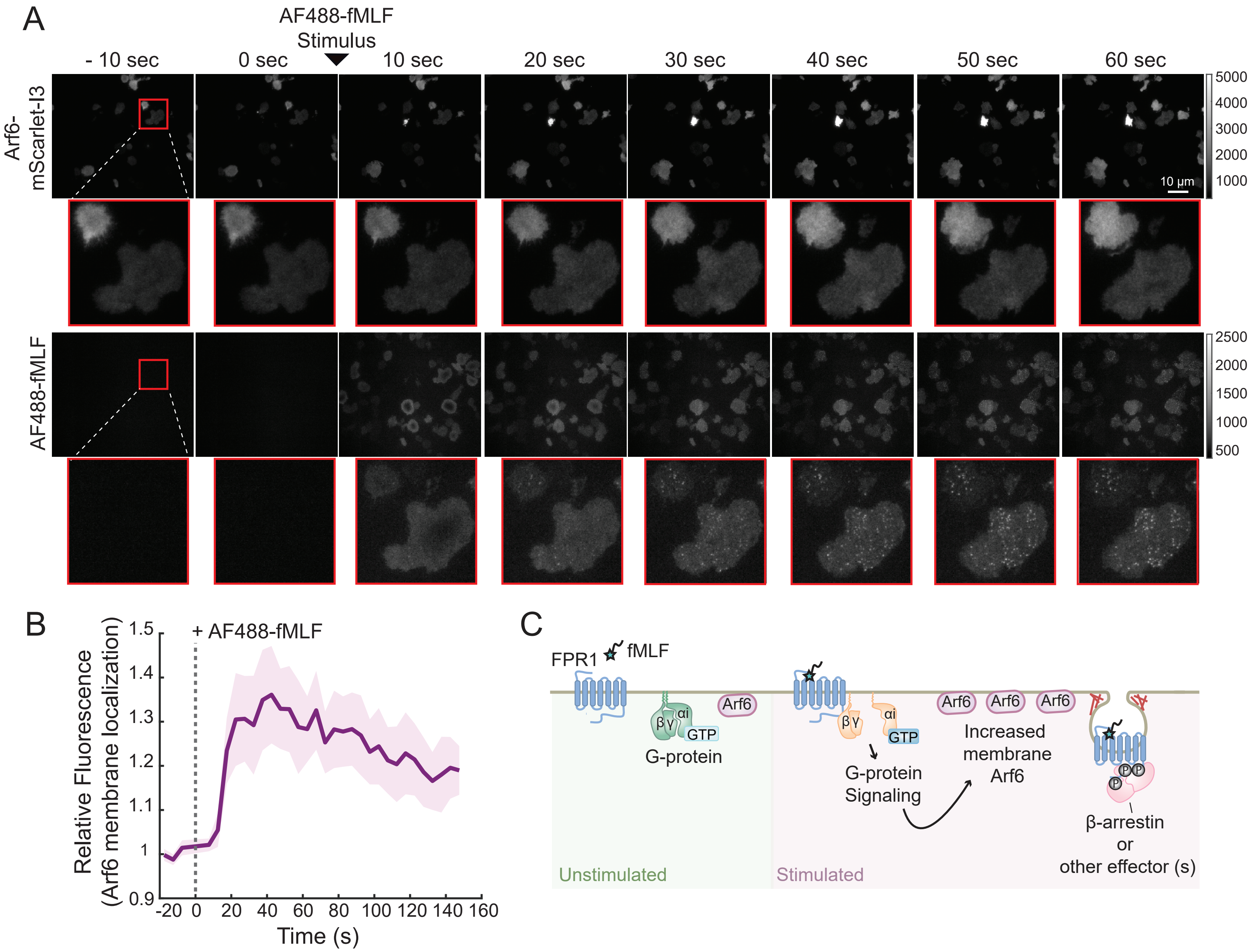

Supplementary Figure 12. FPR1 stimulation enhances Arf6 translocation to the plasma membrane, but Arf6 does not cluster with receptors. (A) Representative images from timeslapse total internal reflection fluorescence imaging of Arf6-mScarlet-I3 before and after AF488-fMLF stimulation (scale bar, 10µm). The red rectangle indicates the magnified site for better visualization of changes at the single cell level. (B) Change in Arf6-mScarlet-I3 fluorescence over time. For quantification, cell masks were created for each image. Fluorescence for the pixels associated with a cell throughout imaging was normalized to the pre-stimulus images and plotted on the y-axis. This approach was used to measure changes in intensity without bias from fluorescence signal changes due to changes in cell morphology or movement during imaging. n=6 wells, >60 cells in total. Data collected on two different days. (C) Schematic showing fMLF-induced plasma membrane recruitment of Arf6 prior to FPR1 internalization.

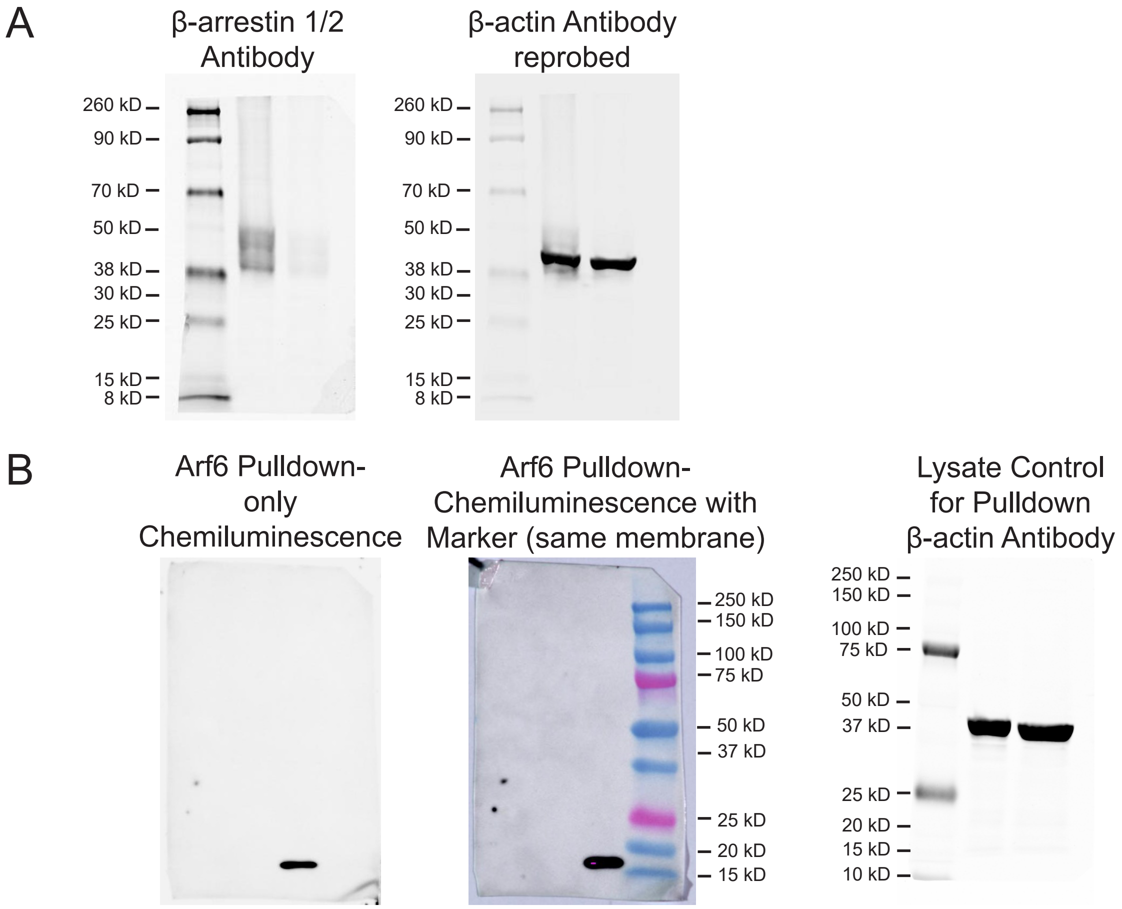

Supplementary Figure 13. Raw Images for Western blots. (A) Raw images for Supplementary figure 4A. (B) Raw images for Supplementary figure 11D.

References:

1. Morgens, D. W., Deans, R. M., Li, A. & Bassik, M. C. Systematic comparison of CRISPR/Cas9 and RNAi screens for essential genes. *Nat*. *Biotechnol*. **34**, 634–636 (2016).
