# Supplementary material for: Parallel CRISPR screens reveal pathways controlling the cell surface levels of the attractant receptor FPR1
Source: Commun Biol. 2026 Mar 25;9:668. doi: 10.1038/s42003-026-09878-3 (PMC13181108; doi:10.1038/s42003-026-09878-3)
Supplement: Supplementary file 11 — Description of Additional Supplementary Files [file 42003_2026_9878_MOESM11_ESM.docx]

**Description of Additional Supplementary File**

File name: Supplementary data 1
Description: Screen scores for all sgRNAs.

File name: Supplementary data 2
Description: Screen scores for all genes.

File name: Supplementary data 3
Description: Negative hits identified in the basal surface FPR1 expression screen.

File name: Supplementary data 4
Description: Positive hits identified in the basal surface FPR1 expression screen.

File name: Supplementary data 5
Description: Negative hits identified in the post-stimulation surface FPR1 expression screen.

File name: Supplementary data 6
Description: Positive hits identified in the post-stimulation surface FPR1 expression screen.

File name: Supplementary data 7
Description: Negative hits identified by the integrated analysis of two screens for FPR1 internalization.

File name: Supplementary data 8
Description: Positive hits identified by the integrated analysis of two screens for FPR1 internalization.

File name: Supplementary data 9
Description: Numerical source data.
