# Supplementary material for: Parallel CRISPR screens reveal pathways controlling the cell surface levels of the attractant receptor FPR1
Source: Commun Biol. 2026 Mar 25;9:668. doi: 10.1038/s42003-026-09878-3 (PMC13181108; doi:10.1038/s42003-026-09878-3)
Supplement: Supplementary file 12 — Reporting summary [file 42003_2026_9878_MOESM12_ESM.pdf]

nature portfolio

Corresponding author(s): Sean Collins, Emel Akdogan

Last updated by author(s): Feb 11, 2026

Reporting Summary

Nature Portfolio wishes to improve the reproducibility of the work that we publish. This form provides structure for consistency and transparency in reporting. For further information on Nature Portfolio policies, see our Editorial Policies and the Editorial Policy Checklist.

Statistics

For all statistical analyses, confirm that the following items are present in the figure legend, table legend, main text, or Methods section.

n/a	Confirmed	
☐	☒	The exact sample size (*n*) for each experimental group/condition, given as a discrete number and unit of measurement
☐	☒	A statement on whether measurements were taken from distinct samples or whether the same sample was measured repeatedly
☐	☒	The statistical test(s) used AND whether they are one- or two-sided<br>*Only common tests should be described solely by name; describe more complex techniques in the Methods section.*
☒	☐	A description of all covariates tested
☐	☒	A description of any assumptions or corrections, such as tests of normality and adjustment for multiple comparisons
☐	☒	A full description of the statistical parameters including central tendency (e.g. means) or other basic estimates (e.g. regression coefficient) AND variation (e.g. standard deviation) or associated estimates of uncertainty (e.g. confidence intervals)
☐	☒	For null hypothesis testing, the test statistic (e.g. *F*, *t*, *r*) with confidence intervals, effect sizes, degrees of freedom and *P* value noted<br>*Give P values as exact values whenever suitable.*
☐	☒	For Bayesian analysis, information on the choice of priors and Markov chain Monte Carlo settings
☒	☐	For hierarchical and complex designs, identification of the appropriate level for tests and full reporting of outcomes
☐	☒	Estimates of effect sizes (e.g. Cohen's *d*, Pearson's *r*), indicating how they were calculated

Our web collection on statistics for biologists contains articles on many of the points above.

Software and code

Policy information about availability of computer code

Data collection	1. Confocal fluorescence microscopy images were acquired using Zeiss ZEN software (3.5).<br>2. Flow cytometry data was collected using BD FACSDiva Sofware.<br>3. Total internal reflections microscopy images were acquired using Micro-Manager (version 2.0) through a Matlab (version 2024b) interface.<br>4. Western blot images were acquired using an Amersham™ Imager 600 (auto exposure) and an Azure Sapphire FL Biomolecular Imager (smart exposure).
Data analysis	Genome-wide screen data was analyzed using custom code written in Matlab version 2024a (Mathworks). The procedures executed by the code is described in the Methods, and the code has been deposited at https://github.com/srcollins/Code-for-Akdogan-et-al.

For manuscripts utilizing custom algorithms or software that are central to the research but not yet described in published literature, software must be made available to editors and reviewers. We strongly encourage code deposition in a community repository (e.g. GitHub). See the Nature Portfolio guidelines for submitting code & software for further information.

Data

Policy information about availability of data

All manuscripts must include a data availability statement. This statement should provide the following information, where applicable:

- Accession codes, unique identifiers, or web links for publicly available datasets
- A description of any restrictions on data availability
- For clinical datasets or third party data, please ensure that the statement adheres to our policy

Sequence data generated from the CRISPR/Cas9 screens and the amplicon sequencing for CRISPR knockout validation are uploaded to the Sequence Read Archive (BioProject ID: PRJNA1246673) and available at http://www.ncbi.nlm.nih.gov/bioproject/1246673. Custom codes used for the analysis of the CRISPR screens are available at https://github.com/srcollins/Code-for-Akdogan-et-al. All data is presented in the figures and supplementary material and is openly available as a GitHub project at https://github.com/emelakdogan/FPR1-Endocytosis-Screen-Paper/.

Research involving human participants, their data, or biological material

Policy information about studies with human participants or human data. See also policy information about sex, gender (identity/presentation), and sexual orientation and race, ethnicity and racism.

Reporting on sex and gender	Consistent with our IRB protocol, no personal information (including sex or gender) was associated with the samples used for our experiments. We did not consider sex or gender because we are studying very basic properties of individual cells.
Reporting on race, ethnicity, or other socially relevant groupings	Consistent with our IRB protocol, no personal information was associated with the samples used for our experiments. We did not consider race, ethnicity, or social variables because we are studying very basic properties of individual cells.
Population characteristics	See above
Recruitment	Healthy donors were recruited through direct person-to-person interactions, with an informed consent process. We do not expect that any biases from recruitment will impact the study results.
Ethics oversight	Institutional Review Board from the University of California, Davis (IORG0000251)

Note that full information on the approval of the study protocol must also be provided in the manuscript.

Field-specific reporting

Please select the one below that is the best fit for your research. If you are not sure, read the appropriate sections before making your selection.

☒ Life sciences ☐ Behavioural & social sciences ☐ Ecological, evolutionary & environmental sciences

For a reference copy of the document with all sections, see nature.com/documents/nr-reporting-summary-flat.pdf

Life sciences study design

All studies must disclose on these points even when the disclosure is negative.

Sample size	Standard sample sizes were used for flow cytometry (as advised by the Flow Cytometry Core at UC Davis and the literature). For CRISPR screens, we used previously established and published sample sizes to achieve ideal coverage (Morgens et al, Nat Commun, 2017).
Data exclusions	Data is only excluded if the control sample data was not as expected due to factors such as flow cytometry laser power differences or technical errors made during preparation of samples.
Replication	To verify reproducibility of data presented, multiple independent experiments were performed on different days. For each replicate, a new batch of differentiated cells were used. CRISPR screens were only done once due to the laborious and costly nature of the process. However, the experiment was done with a large number of cells per guide and ten different guides targeting each gene to ensure confidence in the final dataset. Also, key findings were further confirmed by flow cytometry experiments with multiple repeats. All attempts were successful (unless there was a technical problem on the day see Data exclusions).
Randomization	Randomization is used in tissue culture experiments when possible. Inhibitor pre-treatments and antibody stainings were performed in 6-well and 96-well tissue culture plates. The order of wells for inhibitor or vehicle treatment were randomized between replicates to minimize potential well-to-well variability.
Blinding	Blinding was not relevant in the case of flow cytometry or CRISPR screening experiments, since the data is automatically collected for each sample for a certain number of cells. The gates were set using the control cells, and same gates used for all samples tested on the same day. Samples were blinded for microscopy.

Reporting for specific materials, systems and methods

We require information from authors about some types of materials, experimental systems and methods used in many studies. Here, indicate whether each material, system or method listed is relevant to your study. If you are not sure if a list item applies to your research, read the appropriate section before selecting a response.

Materials & experimental systems

n/a	Involved in the study
☐	☒ Antibodies
☐	☒ Eukaryotic cell lines
☒	☐ Palaeontology and archaeology
☒	☐ Animals and other organisms
☒	☐ Clinical data
☒	☐ Dual use research of concern
☒	☐ Plants

Methods

n/a	Involved in the study
☒	☐ ChIP-seq
☐	☒ Flow cytometry
☒	☐ MRI-based neuroimaging

Antibodies

Antibodies used	We used APC-antiFPR1 (BioLegend catalog # 391610) and FITC-antiFPR1 (Biolegend, catalog # 391604) antibodies for flow cytometry. We used beta-arrestin 1/2 antibody (Cell Signaling catalog #4674), beta-actin antibody (Cell Signaling catalog #4967S), Arf6 antibody (from the Cytoskeleton, Inc kit, catalog number #BK033-S), IRDye® 800CW Donkey anti-Rabbit IgG (LICORbio catalog #926-32213), and Goat anti-mouse HRP conjugated IgG+IgM (Jackson labs catalog #115-035-068) for Western blots.
Validation	We validated the FPR1 antibodies with an FPR1 knockdown cell line, which showed no detectable staining above background.

Eukaryotic cell lines

Policy information about cell lines and Sex and Gender in Research

Cell line source(s)	We used HL-60 cells (PLB-985 sub-line) which were originally obtained from the lab of Dr. Orion Weiner. We also used HEK-293T cells obtained from the ATCC for virus production.
Authentication	HL-60 cells have highly characteristic behaviors, including rapid migration, elongated morphology, and responsiveness to formyl peptides. Cell lines were confirmed based on behavior. We have also validated the identity of these cells by analysis of RNA-seq data and STR profiling. HEK-293T cells were used only for virus production.
Mycoplasma contamination	Cell lines were tested prior to disposal for mycoplasma to confirm the lack of contamination. All cell lines used in the study tested negative.
Commonly misidentified lines (See ICLAC register)	PLB-985 is known to be a misidentified cell line that is actually a sub-line of HL-60 cells. We refer to these cells as HL-60, but we also note in the methods that we used the PLB-985 sub-line. We confirmed that our PLB-985 cells are genetically identical to HL-60 cells by analysis of SNPs. We chose to use this sub-line because we observed better migration in under agarose conditions.

Plants

Seed stocks	*Report on the source of all seed stocks or other plant material used. If applicable, state the seed stock centre and catalogue number. If plant specimens were collected from the field, describe the collection location, date and sampling procedures.*
Novel plant genotypes	*Describe the methods by which all novel plant genotypes were produced. This includes those generated by transgenic approaches, gene editing, chemical/radiation-based mutagenesis and hybridization. For transgenic lines, describe the transformation method, the number of independent lines analyzed and the generation upon which experiments were performed. For gene-edited lines, describe the editor used, the endogenous sequence targeted for editing, the targeting guide RNA sequence (if applicable) and how the editor was applied.*
Authentication	*Describe any authentication procedures for each seed stock used or novel genotype generated. Describe any experiments used to assess the effect of a mutation and, where applicable, how potential secondary effects (e.g. second site T-DNA insertions, mosiacism, off-target gene editing) were examined.*

Flow Cytometry

Plots

Confirm that:

☒ The axis labels state the marker and fluorochrome used (e.g. CD4-FITC).

☒ The axis scales are clearly visible. Include numbers along axes only for bottom left plot of group (a 'group' is an analysis of identical markers).

☒ All plots are contour plots with outliers or pseudocolor plots.

☒ A numerical value for number of cells or percentage (with statistics) is provided.

Methodology

Sample preparation	A neutrophil-like cell line (HL-60, see Eukaryotic cell lines section above for details) was used and maintaied in a 37 degree incubator. Primary human neutrophils are isolated from blood on the day of experiment using EasySep Human Neutrophil Isolation kit (STEMCELL Technologies).
Instrument	BD Canto II (6-color) and BD LSRII (16-color 3-laser) analytic flow cytometers were used for data collection. Beckman Astrios 18-color in BSC was used for cell sorting.
Software	Flow cytometry data was collected using BD FACSDiva Software. Analysis was done using custom MATLAB code, and the code has been deposited at https://github.com/srcollins/Code-for-Akdogan-et-al.
Cell population abundance	Cell sorting was used for cell separation in CRISPR screens. We collected a fixed percentage of cells (30% top and bottom) to ensure representation of control guides to achieve good statistics of enrichment and depletion of guides in different bins. Sample purity is not relevant in these experiments.
Gating strategy	Cells were only gated based on FSC/SSC in flow cytometry experiments to exclude dead cells with small sizes. About 80% of cells were used to collect data in each case. For sorting gates in CRISPR screens, besides the FSC/SSC gate, we also used a gate to ensure all sorted cells are mCherry positive (ensuring expression of a guide), less than 1% of the cells were gated out at this step. All other gates, top 30% at the bottom and top of FITC channel were used for subsequent experimenting for Next Generation Sequencing.

☒ Tick this box to confirm that a figure exemplifying the gating strategy is provided in the Supplementary Information.
