# Supplementary material for: Parallel CRISPR screens reveal pathways controlling the cell surface levels of the attractant receptor FPR1
Source: Commun Biol. 2026 Mar 25;9:668. doi: 10.1038/s42003-026-09878-3 (PMC13181108; doi:10.1038/s42003-026-09878-3)
Supplement: Supplementary file 13 — Transparent Peer Review file [file 42003_2026_9878_MOESM13_ESM.pdf]

Peer Review File

Parallel CRISPR screens reveal pathways controlling the cell surface levels of the attractant receptor FPR1

Corresponding Author: Professor Sean Collins

This file contains all reviewer reports in order by version, followed by all author rebuttals in order by version.

Version 0:

Reviewer comments:

Reviewer #1

(Remarks to the Author)
The authors of the manuscript,titled "Parallel CRISPR screens reveal pathways controlling the cell surface levels of the attractant receptor FPR1"
found that the receptor kinase GRK6 acts in parallel with GRK2 and GRK3 to trigger internalization of FPR1 via β-arrestin-dependent and independent pathways. They used an integrated analysis of two parallel CRISPR/Cas9 screens to identify and classify regulators of FPR1 biogenesis, surface expression, recycling, and endocytosis.They identified the formin mDia1 and the small GTPase Arf6 as specific regulators of FPR1 internalization and confirmed these phenotypes using chemical inhibitors in primary human neutrophils. Furthermore, they found that Arf6 contributes to the β-arrestin-independent signaling pathway.
This is very interesting data that uses new technologies. A complex topic and an important receptor. It is likely that this method can also be used for other GPCR-coupled chemokine receptors.

I have only minor points:
1. Page number/line numbers are missing
2. Figure 1: The authors use AF488-fMLF for stimulation. fMLF is a very small peptide and the modification could change the receptor specificity. Did the authors test, if the modified ligand has the same specificity compared to the original fMLF?
3. Figure 3: How does the inhibition of the b-arrestins instead of the knock out influence FPR1 endocytosis? (e.g. using barbadin)
4. It has been already shown that FPR1 endocytosis/signaling occurs at least in part by a b-arrestin independent signaling, please include Ref.

5. In the methods section: cell maintenance and differentiation: Typos 2x10^5 and 1,5x10^6 instead of 105 and 106
6. Is it possible to anylze with this library also other receptors?

Reviewer #2

(Remarks to the Author)
This manuscript by Akdoğan et al. presents an innovative application of parallel genome-wide CRISPR/Cas9 screens to identify regulators of FPR1 surface expression and internalization in neutrophil-like cells. The authors successfully clarify the roles of multiple GRKs (particularly GRK6) and β-arrestins in FPR1 endocytosis, demonstrating that this receptor uses both β-arrestin-dependent and -independent pathways. The identification of new downstream regulators including the formin mDia1 and small GTPase Arf6 represents a valuable contribution to understanding chemoattractant receptor trafficking mechanisms. The comprehensive dataset generated will serve as a useful resource for future studies on GPCR trafficking. However, several significant methodological limitations require attention, including insufficient validation of screen hits and concerns about the statistical analysis pipeline.
Major comments:
1. While the authors identify 277 positive regulators of FPR1 internalization (i.e., genes whose knockout results in elevated surface FPR1), they provide comprehensive validation for only two hits (mDia1 and Arf6). This raises serious concerns about false discovery rates and the reliability of the screening approach. At minimum, the top 10 hits should be validated using independent methods to establish confidence in the screen results. The lack of broader validation undermines the

utility of the comprehensive dataset that the authors claim will benefit the field.
2. The custom log-likelihood scoring system based on negative binomial modeling is not adequately validated against established methods like MAGeCK or casTLE. The permutation-based p-value calculation needs detailed description, and the authors should demonstrate how their approach compares to standard CRISPR screen analysis tools. Without this comparison, readers cannot assess whether the novel statistical approach introduces systematic biases or improves upon existing methods.
3. The gene knockout validation, such as that of β-arrestin, mDia1 and Arf6, relies only on mRNA levels rather than confirming protein depletion. The authors should employ complementary approaches such as Western blotting or flow cytometry to verify knockout efficiency at the protein level. This is particularly important for genes with post-transcriptional regulation or proteins with long half-lives, where mRNA reduction may not accurately reflect functional knockout.
4. The functional roles of mDia1 and Arf6 in FPR1 internalization remain mechanistically unclear. Do these proteins directly interact with FPR1 or its associated trafficking machinery? The authors should provide protein-protein interaction validation data (e.g., co-immunoprecipitation) to clarify whether these represent direct or indirect regulatory mechanisms.
Minor:
1. Please specify which cell line was used for generating stable CRISPRi knockdowns of the different GRKs (GRK2, GRK3, GRK5, and GRK6), as this information is crucial for reproducibility.
2. In the section “FPR1 uses both β-arrestin-dependent and β-arrestin-independent endocytic pathways,” please correct the incomplete sentence ending with “…the knockouts in pooled cell liFigure S4).”
3. In the section “Genome-wide CRISPR/Cas9 screens reveal regulators of basal FPR1 surface expression,” please add the appropriate figure or table reference to support the conclusion that “targeting mediators of neutrophil differentiation caused decreased basal surface FPR1 levels.”
4. Primary neutrophil isolation and purity assessment details are missing.
5. While the discussion appropriately acknowledges study limitations, it could better address potential off-target effects of the genetic perturbations and chemical inhibitors used, particularly given the reliance on chemical validation approaches.

Version 1:

Reviewer comments:

Reviewer #1

(Remarks to the Author)
The criticisms were addressed satisfactorily.An interesting method that can certainly be used for other receptors in the future.

Reviewer #2

(Remarks to the Author)
The authors have provided additional data and enhanced discussion to fully address all of my concerns. Based on these revisions, I recommend this manuscript for publication.

Open Access This Peer Review File is licensed under a Creative Commons Attribution 4.0 International License, which permits use, sharing, adaptation, distribution and reproduction in any medium or format, as long as you give appropriate credit to the original author(s) and the source, provide a link to the Creative Commons license, and indicate if changes were made.
In cases where reviewers are anonymous, credit should be given to 'Anonymous Referee' and the source.
The images or other third party material in this Peer Review File are included in the article's Creative Commons license, unless indicated otherwise in a credit line to the material. If material is not included in the article's Creative Commons license and your intended use is not permitted by statutory regulation or exceeds the permitted use, you will need to obtain permission directly from the copyright holder.
To view a copy of this license, visit https://creativecommons.org/licenses/by/4.0/

We appreciate the reviewers' comments. In this revised manuscript, we have addressed all concerns raised, which has strengthened our study.

In summary,

1) We have performed new experiments and added 5 new supplementary figures and 2 new supplementary subfigures addressing the reviewers comments (attached and discussed in detail below).
2) We have performed new analysis using previously published CRISPR screening data to validate and compare our analysis strategy to the existing methods (casTLE) and added a new supplementary figure: Supplementary figure 6 in response to Reviewer #2, Major comment 2.
3) We added casTLE scores at the gene level to an existing table for gene level scores, (now called Supplementary Table 2) and scores (p values, Effect and CLO scores) at the single guide level as a new table (Supplementary Table 1) in our revised manuscript.
4) We have made additions and corrections to the text in response to reviewers' comments, which are highlighted in blue in the revised manuscript and copied below to the relevant questions.
5) With the addition of new supplementary figures and tables, the numbers and subfigure letter calls are now changed. All figure and table calls are updated in the revised manuscript. Only new figures and tables are highlighted in blue.
6) With addition of the constructs used in the new experiments, we updated the list of oligonucleotide sequences given in Supplementary Table 9
7) We changed our subsection titled 'Statistics' to 'Statistics and Reproducibility' as suggested by the Revised Manuscripts Submission Checklist, and we have added more details. All additions to the text are marked in blue.
8) A recent paper published in *Science* last month showed that FPR1 signaling plays a crucial role in sustained neuroinflammation in multiple sclerosis (MS), and FPR1 antagonists can slow down progression of MS in mouse models[1]. This work further supports the significance of studying FPR1 regulation. Therefore, we added a new reference in the main text where we mention FPR1 as a therapeutic target without any change in the text: "... FPR1 has been explored as a therapeutic target to limit neutrophil-dependent damage in inflammation ..."

Please find below our detailed responses to each reviewer comment. We have left the reviewer comments in black, and we have included our replies in blue text.

Reviewers' comments:

Reviewer #1 (Remarks to the Author):

The authors of the manuscript,titled "Parallel CRISPR screens reveal pathways controlling the cell surface levels of the attractant receptor FPR1" found that the receptor kinase GRK6 acts in

parallel with GRK2 and GRK3 to trigger internalization of FPR1 via β-arrestin- dependent and independent pathways. They used an integrated analysis of two parallel CRISPR/Cas9 screens to identify and classify regulators of FPR1 biogenesis, surface expression, recycling, and endocytosis. They identified the formin mDia1 and the small GTPase Arf6 as specific regulators of FPR1 internalization and confirmed these phenotypes using chemical inhibitors in primary human neutrophils. Furthermore, they found that Arf6 contributes to the β-arrestin-independent signaling pathway.
This is very interesting data that uses new technologies. A complex topic and an important receptor. It is likely that this method can also be used for other GPCR-coupled chemokine receptors.

I have only minor points:
1. Page number/line numbers are missing

We added page and line numbers to the revised manuscript.

2. Figure 1: The authors use AF488-fMLF for stimulation. fMLF is a very small peptide and the modification could change the receptor specificity. Did the authors test if the modified ligand has the same specificity compared to the original fMLF?

We thank the reviewer for their concern about the specificity of the fluorescently tagged ligand used in our study. Fluorescently tagged formyl peptides (e.g. FLPEP, FNLFNYK-FITC, and AF488-fMLF) have long been used to study neutrophil signaling and behavior. AF488-fMLF and another fluorescently tagged formyl peptide, FNLFNYK-FITC, were previously published in another study that investigated FPR1 trafficking during chemotaxis. Using microscopy, Subramanian et al[2] showed that FPR1 sgRNA expressing HL-60 cells do not internalize FNLFNYK-FITC, indicating addition of a fluorescent tag did not have a significant effect on the specificity of the ligand for FPR1. We added a reference to this paper in Line 149.

To ensure the specificity of AF488-fMLF for FPR1 in our hands, we have used this peptide to stain surface FPR1 in differentiated HL-60 cells that express only dead Cas9 (dCas9) or dCas9 with an FPR1 targeting sgRNA (see the figure below). Staining is done on ice to minimize receptor internalization for 1 minute at 100 nM final concentration in the buffer. Only cells expressing FPR1 were stained with AF488-fMLF, and signal from FPR1 knockdown cells looked similar to unstained control cells. This result confirms the specificity of AF488-fMLF for FPR1 in our experiments.

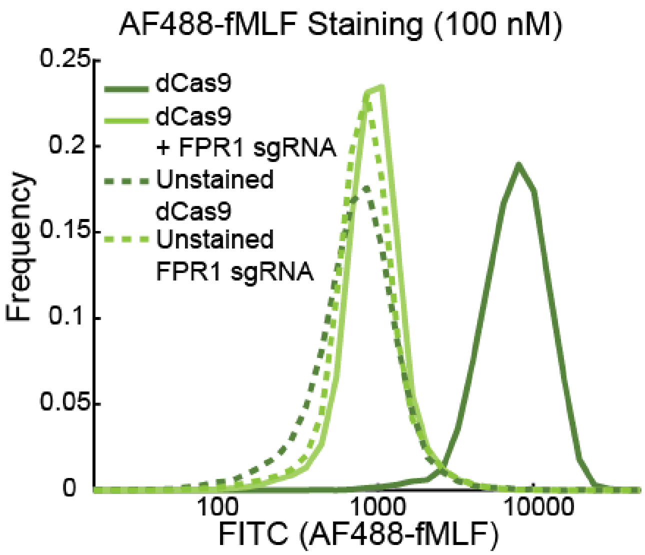

3. Figure 3: How does the inhibition of the b-arrestins instead of the knock out influence FPR1 endocytosis? (e.g. using barbadin)

Below, we attached a figure showing Barbadin has no significant effect on FPR1 internalization, while it slightly elevates the basal surface FPR1 levels. Barbadin is characterized as blocking the interaction between β-arrestins and AP-2 without a significant effect on the receptor- β-arrestin interaction[3]. Our screens did not identify AP-2 components or clathrin as regulators of FPR1 surface levels. Therefore we think this inhibitor may block some arrestin functions but not their role in FPR1 endocytosis. Slight elevation in FPR1 basal levels may point to a role for this complex in constitutive FPR1 recycling in unstimulated cells. We did not include this figure in our revised manuscript, since our data did not support a role for the β-arrestin/AP2 complex in FPR1 endocytosis.

In the figure below, we pre-incubated cells with 50 µM Barbadin (MedChemExpress, catalog #HY-119706) or vehicle for 30 min at 37 deg. We then added 100 nM fMLF or completed media and incubated cells 10 min at 37 deg. Following stimulation, we washed away the unbound ligand and performed antibody staining for surface FPR1 and flow cytometry measurements as described in our manuscript.

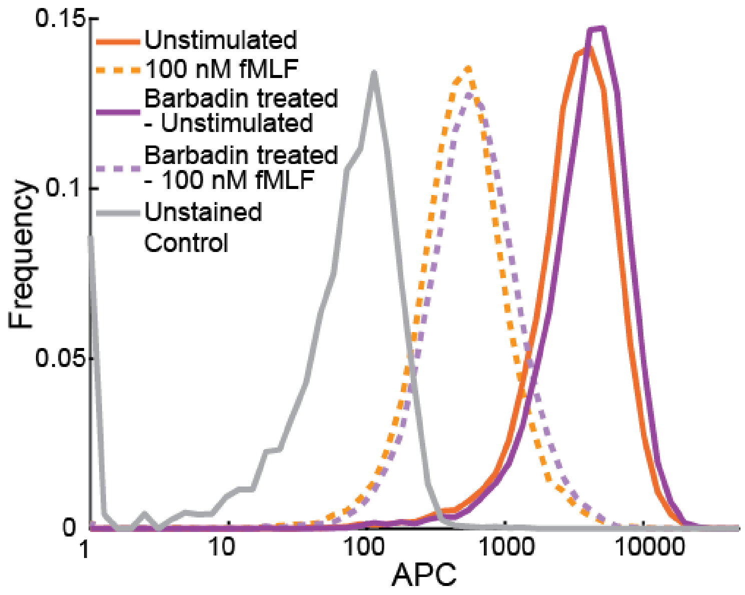

4. It has been already shown that FPR1 endocytosis/signaling occurs at least in part by a b-arrestin independent signaling, please include Ref.

We thank the reviewer for their comment. We now added a reference[4] to the topic sentence in the Results section under 'FPR1 uses both β-arrestin-dependent and β-arrestin-independent endocytic pathways' subtitle (Line 216): "Although β-arrestins control the endocytosis of many GPCRs, FPR1 can internalize in a β-arrestin-independent manner, at least in heterologous cell types." The following sentences in this section already had references to the specific research papers where β-arrestins' role in FPR1 internalization is investigated in different cell types.

5. In the methods section: cell maintenance and differentiation: Typos 2x10^5 and 1,5x10^6 instead of 105 and 106

Thank you for catching this mistake. We have made the necessary corrections.

6. Is it possible to analyze with this library also other receptors?

We thank the reviewer for raising this question. Yes, this strategy should work similarly for other receptors. Our FACS-based cell selection strategy is optimized for receptors with high internalization capacity. For receptors that are internalized at high levels, similar screens could be performed with different ligand and antibody pairs. In our revised manuscript, we added a comment on this to the Discussion section under 'Further Implications' subtitle (Lines 620-622): "Towards this end, we note that our strategy using paired FACS-based screens could easily be adapted to investigate the cell surface regulation of other receptors."

Reviewer #2 (Remarks to the Author):

This manuscript by Akdoğan et al. presents an innovative application of parallel genome-wide CRISPR/Cas9 screens to identify regulators of FPR1 surface expression and internalization in neutrophil-like cells. The authors successfully clarify the roles of multiple GRKs (particularly GRK6) and β-arrestins in FPR1 endocytosis, demonstrating that this receptor uses both β-arrestin-dependent and -independent pathways. The identification of new downstream regulators including the formin mDia1 and small GTPase Arf6 represents a valuable contribution to understanding chemoattractant receptor trafficking mechanisms. The comprehensive dataset generated will serve as a useful resource for future studies on GPCR trafficking. However, several significant methodological limitations require attention, including insufficient validation of screen hits and concerns about the statistical analysis pipeline.

Major comments:

1. While the authors identify 277 positive regulators of FPR1 internalization (i.e., genes whose knockout results in elevated surface FPR1), they provide comprehensive validation for only two hits (mDia1 and Arf6). This raises serious concerns about false discovery rates and the reliability of the screening approach. At minimum, the top 10 hits should be validated using independent methods to establish confidence in the screen results. The lack of broader validation undermines the utility of the comprehensive dataset that the authors claim will benefit the field.

We thank the reviewer for their comment. We agree that it is important to validate a larger number of hits to support the biological relevance of the hits identified for different phenotypes. To address this point, we selected the three strongest hits for each hit category from our primary screens, as well as the top three for each integrated analysis hit category (excluding genes that were strong hits for a primary screen). This gave a list of 12 genes, as a few genes were in the top three for more than one category. We generated CRISPRi knockdowns with two independent guides for each of these genes and measured their phenotypes in low throughput. These experiments largely confirmed the observed phenotypes in the CRISPR screens. We have added a new supplementary figure (Supplementary Figure 7) and the corresponding text (Lines 318-328) in the revised manuscript:

“To validate a subset of hits directly, we selected the three strongest hits for each hit category from our primary screens (low or high basal surface FPR1 and low or high post stimulation surface FPR1), as well as the top three for each integrated analysis hit category (excluding genes that were strong hits for a primary screen). This gave a list of 12 genes (CSNK1A1, ZBTB7A, MLST8, CCDC22, COMMD5, PTEN, SLC35A2, SIK3, GALE, RAB11A, EXOC2, RGL2), as a few genes were in the top three for more than one category (Supplementary Figure 7A-D). We generated CRISPRi knockdowns with two independent guides for each of these genes and measured the effects on FPR1 surface expression levels before and after fMLF stimulation (Supplementary Figure 7A and B). The results were largely consistent with the

results from our screen, including apparent defects in internalization for knockdowns of CCDC22, COMMD5, and SLC35A (Supplementary Figure 7C).”

Furthermore, in response to the reviewer’s concern about hit reliability and the false discovery rate, we note that our screens utilize 10 guides for each gene, and our analysis accounts for empirical variability of phenotypes for more than 10,000 control guides, providing strong statistical data for estimating the rate of false positives. Further analysis we performed to validate our custom log-likelihood scoring system (explained in detail in response to the next comment) also supports the power of our approach with comparable false discovery rates to existing analysis strategies.

New Supplementary Figure 7: **Validation of top hits across different phenotypes from Surface FPR1 and Post-stim Surface FPR1 screens.** Differences (log2 fold change, log2FC) in surface FPR1 levels are shown in (A) unstimulated and (B) stimulated (10 min, 100 nM fMLF) cells with CRISPRi knockdown of 12 genes (CSNK1A1, ZBTB7A, MLST8, CCDC22, COMMD5, PTEN, SLC35A2, SIK3, GALE, RAB11A, EXOC2, RGL2) with two independent guides per gene (indicated by g1 and g2). (C) Percentage of surface FPR1 remaining after stimulation are shown for genes identified as potential regulators of FPR1 internalization through integrated analysis of two screens. n=6 biological replicates. (D) A heatmap of log P-scores obtained from CRISPR screens is shown for the 12 genes tested. Blue represents a phenotype with low surface FPR1 and yellow represents high surface FPR1.

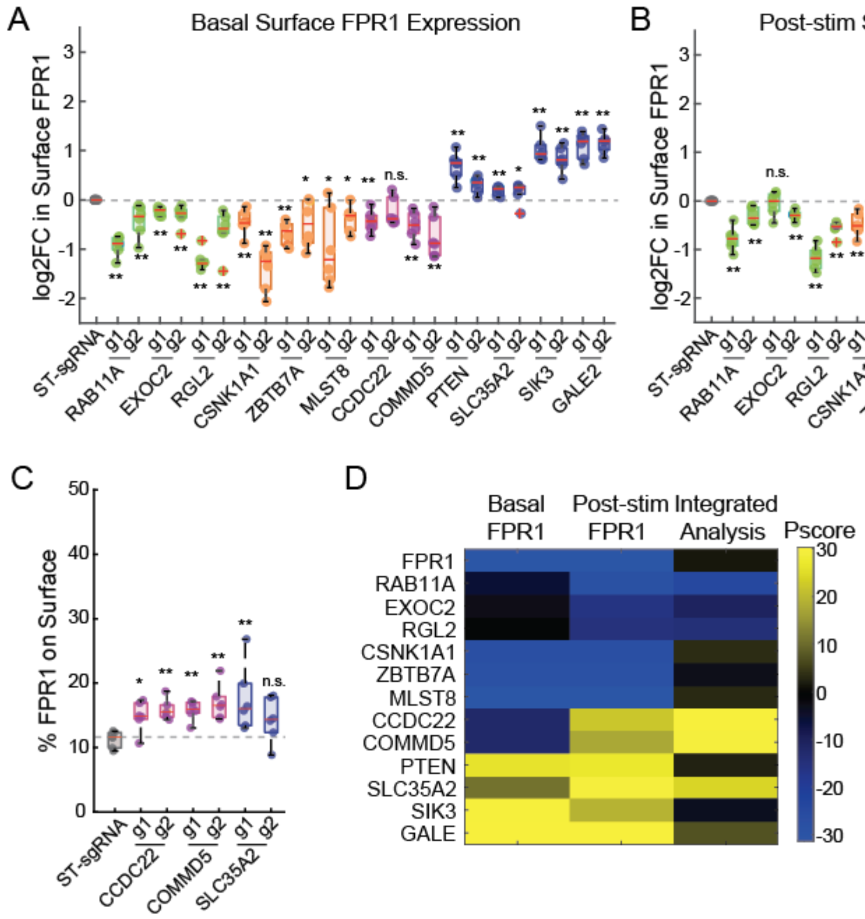

2. The custom log-likelihood scoring system based on negative binomial modeling is not adequately validated against established methods like MAGeCK or casTLE. The permutation-based p-value calculation needs detailed description, and the authors should demonstrate how their approach compares to standard CRISPR screen analysis tools. Without this comparison, readers cannot assess whether the novel statistical approach introduces systematic biases or improves upon existing methods.

We agree that validation of our scoring approach relative to an established approach is important to give readers confidence in our results, and we thank the reviewer for this request. We have added a new supplementary figure (Supplementary Figure 6) showing a comparison of our log-likelihood scoring system with the casTLE approach, including a direct comparison using the same data set that was used to compare casTLE to MAGeCK and other approaches in the initial methods paper[5] describing the casTLE approach.

We created and used our new approach in large part because it provides a natural way to handle the 4-sample comparison to identify genes specifically impacting internalization and recycling. We did not feel that there was an existing method well-suited for this purpose. To

validate our approach, we focused on comparing it to the well-established casTLE approach on two-sample comparisons.

First, we apologize for the lack of clarity about our p-value calculations. Our procedure was accurately described in our methods section. However, we had erroneously described it as "permutation-based" in the main text. Our p-value comparisons are empirical, based on the rank order of guide scores relative to the control guides. We compute gene-level p-values using an established approach to combine p-values from independent measurements. We have corrected the main text (Lines 270-271) to more accurately describe our approach.

Second, we have added a direct comparison of our approach with the casTLE scoring approach. We computed casTLE scores for our two primary screens (Surface and Post-Stim) for comparison with our new scores. Our scores are strongly correlated with the casTLE scores (Supplementary Figure 6A and B), indicating general agreement between the two methods.

To validate the accuracy of our approach, we also computed CLO scores and Log P scores using our approach for the gene essentiality screens published in Morgens et al[5]. In the Morgens paper, these two replicate CRISPR screens were used to assess the casTLE approach relative to MAGeCK, HiTSelect, RIGER, and other methods using Receiver Operator Curves (ROC) based on a previously annotated "gold standard" set of true positive and true negative "essential genes". In that comparison, the casTLE approach performed approximately equally to MAGeCK and HiTSelect, and all three outperformed several other methods including RIGER. We computed ROC curves on the same data set using our CLO and Log P scores. We found that our method performed approximately equally well as the casTLE approach, with a very slightly higher area under the curve values (Supplementary Figure 6C and D).

Finally, we compared both casTLE and our scores for reproducibility over the two replicate screens from Morgens et al. Both approaches gave strongly correlated scores for the two replicate screens, although the correlations were tighter for our CLO and Log P scores (Supplementary Figure 6E-G).

Together, these results validate the CLO and Log P scores for efficiently identifying hits from CRISPR screens. We have also included the casTLE scores in our supplementary data set (Supplementary Table 2), so that readers can check the corresponding scores for corroboration.

We added the following paragraph in the main text (Lines 278-285): "We validated our log-likelihood scoring system against the established casTLE method for our two sample comparisons, and found that casTLE scores strongly correlate with log P-scores (Supplementary Figure 6A and B, Supplementary Table 2). Moreover, we used our method to analyze two CRISPR gene essentiality screens that were previously used to compare casTLE to MAGeCK and other approaches. Our approach accurately identified essential genes with an area under the receiver operating characteristic (ROC) curve slightly higher than that of the casTLE approach (Supplementary Figure 6C and D). Our scores for these screens were also strongly correlated with the casTLE scores (Supplementary Figure 6E, F, and G)."

New Supplementary Figure 6: **Validation of the log-likelihood scoring system by comparison to the established casTLE analysis.** Comparison of log P-scores and casTLE scores across all genes for single screens: (A) Surface Screen and (B) Post-stim Screen. Reanalysis of published gene essentiality screens using our log likelihood scoring (log P, CLO) method and its comparison to casTLE analysis. ROC curves for identifying essential genes using these methods were calculated for screen replicate 1 (C) and replicate 2 (D). True and false positive rates were calculated as described previously. Distribution and consistency of casTLE, log P and CLO scores across the two replicate gene essentiality screens are shown in scatter plots for each scoring method (E-G).

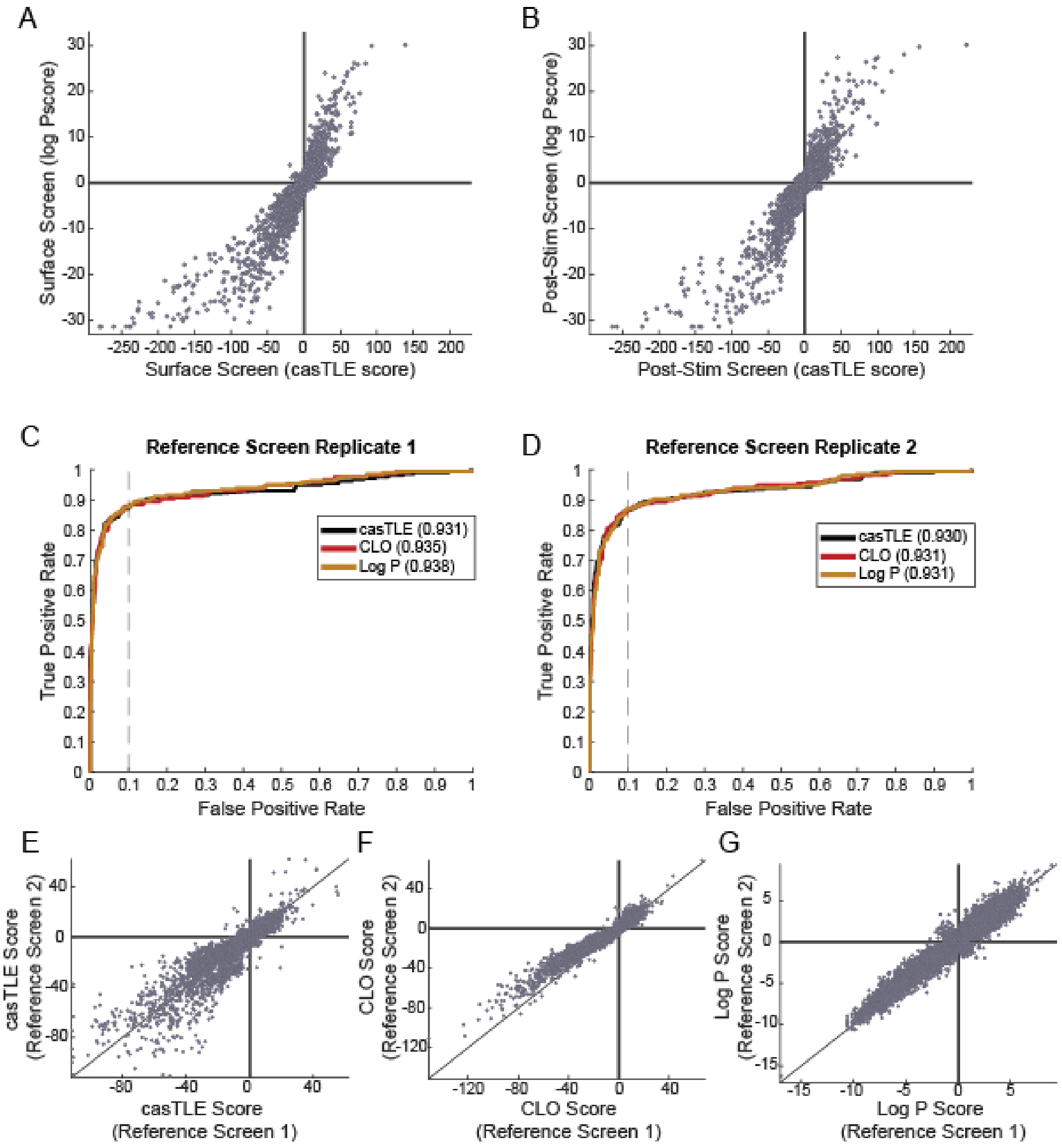

3. The gene knockout validation, such as that of β-arrestin, mDia1 and Arf6, relies only on mRNA levels rather than confirming protein depletion. The authors should employ complementary approaches such as Western blotting or flow cytometry to verify knockout efficiency at the protein level. This is particularly important for genes with post-transcriptional regulation or proteins with long half-lives, where mRNA reduction may not accurately reflect functional knockout.

To confirm the efficiency of CRISPR editing at the protein level, we added western blots on total cell lysates for β-arrestins. For Arf6, we tried multiple antibodies and all had nonspecific staining with a similar or brighter signal to the Arf6 band, leaving some uncertainty about specificity. Therefore, we used GGA3-agarose beads to first collect Arf proteins from the total cell lysate (Cytoskeleton Inc, kit product #BK033-S). We then subjected the eluate to western blot using an Arf6 antibody. For both β-arrestins and Arf6, we found that the relevant proteins were absent in the knockout cell lines. We added these results to supplementary material as Supplementary Figure 4A (for β-arrestin double knockout) and Supplementary Figure 11D (for Arf6 knockout). We note that our manuscript also includes amplicon sequencing as an alternative approach to validate these knockout cell lines. In our initial manuscript, we did not have any experiments with an mDia1 knockout, so we think the question about a protein level validation for its knockout was a confusion.

In the main text, we added Lines 229-230: ”We confirmed knockout efficiency at the protein level by 229 Western blot using a β-arrestin1/2 antibody (Supplementary Figure 4A).” and Lines 492-493: “We further confirmed that Clone 9 does not have 492 any detectable Arf6 protein by Western blot (Supplementary Figure 11D).”

Revised Supplementary Figure 4: **Pooled β-arrestin DKO is efficient and has a similar endocytosis phenotype to individual clones.** (A) Western blotting to confirm β-arrestin 1/2 double knockout efficiency. Cell lysates were used to assess β-arrestin levels. Staining for loading control (β-actin) was done on the same membrane. Image is representative of n=3 biological replicates. Clonally selected β-arrestin double CRISPR knockout cells are compared to pooled double β-arrestin knockout cell lines using the FPR1 endocytosis assay. Cells were untreated or stimulated with 1 µM fMLF. (B) A representative plot showing fMLF-induced decrease in surface FPR1 in the control sgRNA expressing cells. (C) Pooled double β-arrestin knockout cells have a smaller shift after fMLF stimulation. (D) Representative plots obtained from twelve clonally selected β-arrestin double knockout cell lines. Phenotypes observed in clonally selected and pooled knockout cell lines resemble each other.

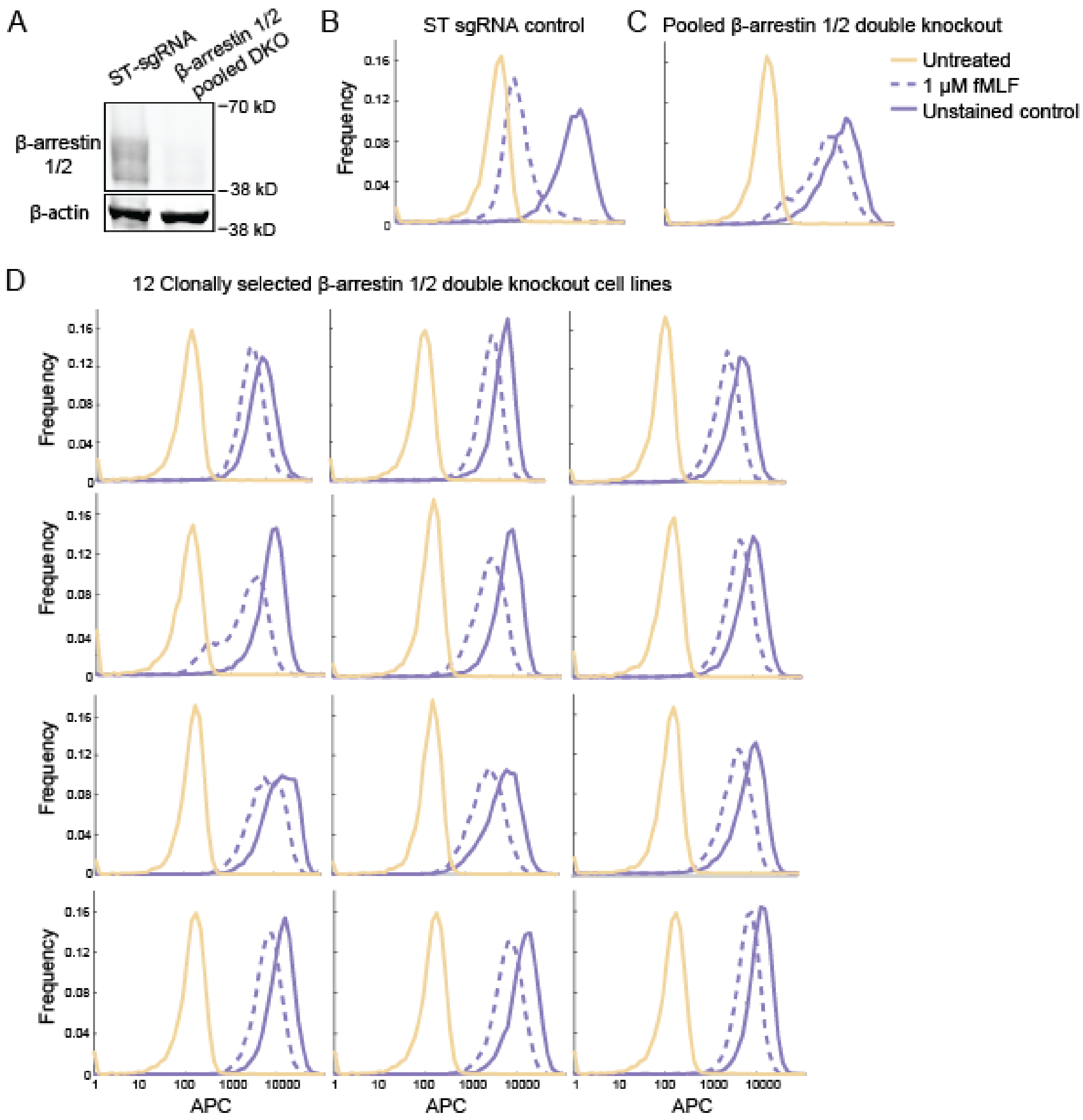

Revised Supplementary Figure 11: **Confirmation of Arf6 knockout efficiency.** Sequences with read numbers greater than 1% of the total reads for each sample are presented. (A) ARF6 sequences for ST-sgRNA expressing cells, pooled ARF6 sgRNA#1, and Clones 1 and 4. (B) ARF6 sequences for ST-sgRNA expressing cells, pooled ARF6 sgRNA#2, and Clone 9. The targeted sequence by the sgRNA is highlighted in gray. We detected a frequent base variant in the control cell sequences from the reference sequence (G>A, highlighted in green), and we refer to this variant as the alternative wild-type (alt wt). Mutant percentage is calculated by subtracting the percentage of reads that contain the intact control sequence (wild-type or alt wt) from 100. (C) Western blotting to confirm Arf6 knockout efficiency at the protein level. We pulled down Arf GTPases in total cell lysates of ST-sgRNA expressing control cells and Arf6 knockout cells (Clone 9) using the Arf6 Pull-down Kit from Cytoskeleton Inc (see Methods for details). A sample from normalized total lysate was used to access total protein levels. All samples, pulldown and lysate, were run on the same gel and transferred to the same membrane, but they

were probed with different antibodies (Arf6 antibody for pulldown samples and β-actin antibody for total lysate controls). Image is representative of n=3 biological replicates. (D) qPCR to detect presence of ARF6 mRNA in control sgRNA expressing and clonally selected ARF6 sgRNA expressing cells.

A

Reference Sequence CCCCACG-AGATCCAGGAGAAACTGGG

Cell Line	# of Reads	Sequence	Type of Mutation
ST sgRNA	23168	CCCCACA-AGATCCAGGAGAAACTGGG	alt wt
0.42% mutant	8153	CCCCACG-AGATCCAGGAGAAACTGGG	wild type
ARF6 sgRNA#1	18145	CCCCACA-AGATCCAGGAGAAACTGGG	alt wt
Pool KO	1547	CCCCACGAAGATCCAGGAGAAACTGGG	insertion, frameshift
95.15% mutant	1504	CCCCAC---GATCCAGGAGAAACTGGG	deletion
	1271	CC----------CCAGGAGAAACTGGG	deletion, frameshift
ARF6 sgRNA#1	16455	CCCCACA-AGATCCAGGAGAAACTGGG	alt wt
Clone #1	9209	CC----------CCAGGAGAAACTGGG	deletion, frameshift
96.04% mutant			
ARF6 sgRNA#1	19867	CCCCACA-AGATCCAGGAGAAACTGGG	alt wt
Clone #4	6836	CCCCACGAAGATCCAGGAGAAACTGGG	insertion, frameshift
92.68% mutant			

B

Reference Sequence TCAAGTTCAACGTATGGGAT-GTGGGC

ST sgRNA	40494	TCAAGTTCAACGTATGGGAT-GTGGGC	wild type
0.28% mutant	40157	TCAAGTTCAACATATGGGAT-GTGGGT	alt wt
	1885	TCAAGTTCAACGTATGGGAT-GTGGGC	wild type
	1759	TCAAGTTCAACGTATGGGAT-GTGGGC	wild type
ARF6 sgRNA#2	19740	TCAAGTTCAACGTATGGGA--------	deletion, frameshift
Pool KO	8165	TCAAGTTCAACGTAT----T-GTGGGC	deletion, frameshift
99.20% mutant	6691	TCAAGTTCAACATGTG--------GGT	deletion, frameshift
	6127	TCAAGTTCAACATATG--------GGT	deletion, frameshift
ARF6 sgRNA#2	37695	---------------------------	deletion, frameshift
Clone #9	14408	TCAAGTTCAACATATGGGATTGTGGGT	insertion, frameshift
99.29% mutant	2524	TCAAGTTCAACATATG------TGGGT	deletion
	2090	TCAAGTTCAACATATG--------GGT	deletion, frameshift

C

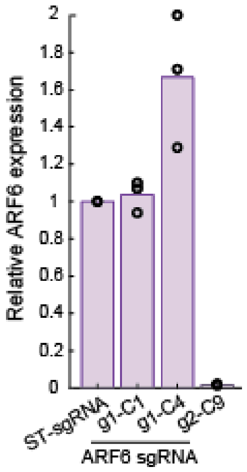

D

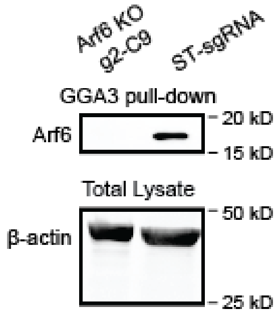

We added details on how we performed the western blots in the Methods section under the 'Western Blotting' subtitle: "Differentiated HL-60 cells ($4×10^7$ for Arf6 and $2×10^7$ for β-arrestin samples) were lysed in 25mM Tris pH 7.2, 5mM MgCl2, 0.15M NaCl, and 1% Igepal (Cytoskeleton Inc, Part # A6LB). Cell lysates were incubated on ice for 5 min and clarified by centrifugation (10,000 rpm, 6 min, 4°C). Aliquots of each lysate were used to normalize total amount protein in each sample (input). Samples were then incubated with 40 µg of GST-GGA3 immobilized on glutathione-Sepharose (Cytoskeleton Inc, Arf6 Pull-down Activation Assay Biochem Kit, product #BK033-S) for 1 h at 4°C to concentrate Arf proteins. The pellets were washed three times with 600 µl lysis buffer. Proteins were eluted by boiling in 30 µl Leammli sample buffer for 5 min. 20 µL of each Arf pulldown sample and total cell lysates (to control for total protein levels) were loaded to precast protein gels to perform western blot.

To test β-arrestin double knockout efficiency, total cell lysates of ST-sgRNA expressing control and β-arrestin double knockout cells were obtained as described above. 20 uL of normalized protein samples were loaded into a 10% precast gel (Bio-Rad, catalog #4561033). All western blots were run at 100V for around 75 min. Proteins then were transferred to the PVDF membrane at 75V for 50 min at 4°C. Membranes were blocked using 5% dry milk (w/V, RPI Research Products #M17200-500.0) solution in Tris-buffered saline (20 mM Tris-HCl, pH 7.4, and 150 Mm NaCl) containing 0.05% Tween (TBST). Primary antibody incubations were done in blocking solution at 4°C overnight with agitation (Arf6 antibody from Cytoskeleton Inc catalog #, β-arrestin 1/2 antibody from Cell Signaling catalog #4674, β-actin antibody from Cell Signaling catalog #4967S). Secondary antibody treatments were performed in blocking solution at room temperature for 1 hour (IRDye® 800CW Donkey anti-Rabbit IgG, product #926-32213, at 1:2500, from LICORbio and Goat anti-mouse HRP conjugated IgG+IgM from Jackson labs catalog #115-035-068). After each antibody incubation, the membranes were washed 3 times with TBST and once with TBS. Before the staining for the loading control (β-actin), the membranes were washed with TBST (0.05% Tween) 5 times for 10 min. Arf6 signal was detected using the SuperSignal™ West Dura Extended Duration Substrate (Thermo Scientific, catalog # 37071). Chemiluminescence is detected with an Amersham™ Imager 600 (auto exposure). β-arrestin and β-actin (control for total protein levels) were detected using a Sapphire FL Biomolecular Imager using smart exposure (Azure Biosciences). Sizes of the protein of interest were confirmed using Chameleon® Duo Pre-stained (Li-Cor, catalog #928-60000) or Precision Plus Dual Color (Bio-Rad, catalog #1610374) protein ladders. ImageJ (Fiji, version 2.14.0) software with QuickFigures plugin was used to cut and adjust images to make figures. Raw images for Western blots are provided in Supplementary Figure 13."

New Supplementary Figure 13: **Raw Images for Western blots.** (A) Raw images for Supplementary figure 4A. (B) Raw images for Supplementary figure 11.

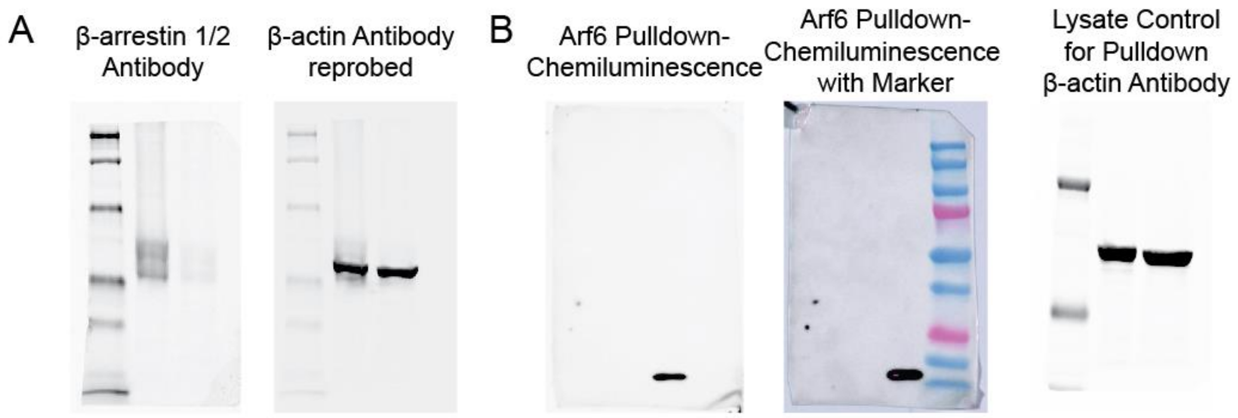

4. The functional roles of mDia1 and Arf6 in FPR1 internalization remain mechanistically unclear. Do these proteins directly interact with FPR1 or its associated trafficking machinery? The authors should provide protein-protein interaction validation data (e.g., co-immunoprecipitation) to clarify whether these represent direct or indirect regulatory mechanisms.

We appreciate the reviewer's questions on the mechanistic roles of mDia1 and Arf6 in FPR1 internalization. Although these proteins have been studied for many years in different contexts, their association with GPCR regulation or endocytosis are still not clear. The literature suggests roles for Arf6 in activation of lipid modifying enzymes and actin cytoskeleton organization, both of these processes are proposed to facilitate endocytosis. mDia1 promotes actin polymerization, which is crucial for regulation of different cell processes.

We were concerned that the specific co-IP approach suggested by the reviewer might yield results that would be difficult to interpret for several reasons. First, we did not have a highly specific antibody for Arf6 (as explained in response to Major Comment #3 above). Second, immunoprecipitations of transmembrane proteins such as FPR1 can be challenging biochemically, especially for isolating and preserving transient interactions. Finally, we expect that both Arf6 and mDia1 are likely to be recruited to the plasma membrane in response to FPR1 activation, and this recruitment could be difficult to distinguish from direct interaction with the receptor. For these reasons, we pursued an imaging-based approach instead. We focused on Arf6, since Arf6 is known to cluster at sites of clathrin-mediated endocytosis in some circumstances[6].

We generated a new stable cell line expressing Arf6-mScarlet-I3 and investigated if Arf6 co-localizes with the fluorescent FPR1 ligand AF488-fMLF during endocytosis using TIRF microscopy. After addition of AF488-fMLF, we rapidly saw the appearance of AF488-fMLF clusters, which likely reflect sites of clustering and endocytosis of FPR1. We did not observe Arf6-mScarleti3 clusters upon AF488-fMLF stimulation or any apparent colocalization of Arf6 and AF488-fMLF. However, we observed a significant increase in Arf6 localization to the plasma membrane upon stimulation. This is consistent with the previously published biochemical results and likely reflects stimulus-induced activation of Arf6. Our results suggest that the role of Arf6

may be indirect, for example possibly through regulating PIP2 lipids. However, resolving the mechanistic roles of these proteins in FPR1 endocytosis will require future in-depth studies.

In the main text, we added Lines 499-509: “To gain more insight into Arf6’s involvement in FPR1 internalization, we investigated the membrane dynamics of a fluorescently tagged Arf6 construct in dHL-60s upon stimulation with the fluorescent ligand (AF488-fMLF) using total internal reflection fluorescence (TIRF) microscopy (Supplementary Figure 12). While AF488-fMLF rapidly formed clusters (~10-30 sec), likely reflecting the sites of FPR1 clustering and internalization, Arf6-mScarlet-I3 did not form apparent clusters that colocalize with the AF488 signal (Supplementary Figure 12A). This observation is in contrast to the clustering of Arf6 at sites of clathrin-mediated endocytosis in HeLa cells. However, we did observe an increase in Arf6 localization to the plasma membrane after stimulation (Supplementary Figure 12B and C). This suggests that Arf6 is activated downstream of FPR1, consistent with biochemical results in a previous study, although its precise mechanistic role in this internalization pathway remains unclear.”

New Supplementary Figure 12: **FPR1 stimulation enhances Arf6 translocation to the plasma membrane, but Arf6 does not cluster with receptors.** (A) Representative images from timeslapse total internal reflection fluorescence imaging of Arf6-mScarlet-I3 before and after AF488-fMLF stimulation. The red rectangle indicates the magnified site for better visualization of changes at the single cell level. (B) Change in Arf6-mScarlet-I3 fluorescence over time. For quantification, cell masks were created for each image. Fluorescence for the pixels associated with a cell throughout imaging was normalized to the pre-stimulus images and plotted on the y-axis. This approach was used to measure changes in intensity without bias from fluorescence signal changes due to changes in cell morphology or movement during imaging. n=6 wells, >60 cells in total. Data collected on two different days.

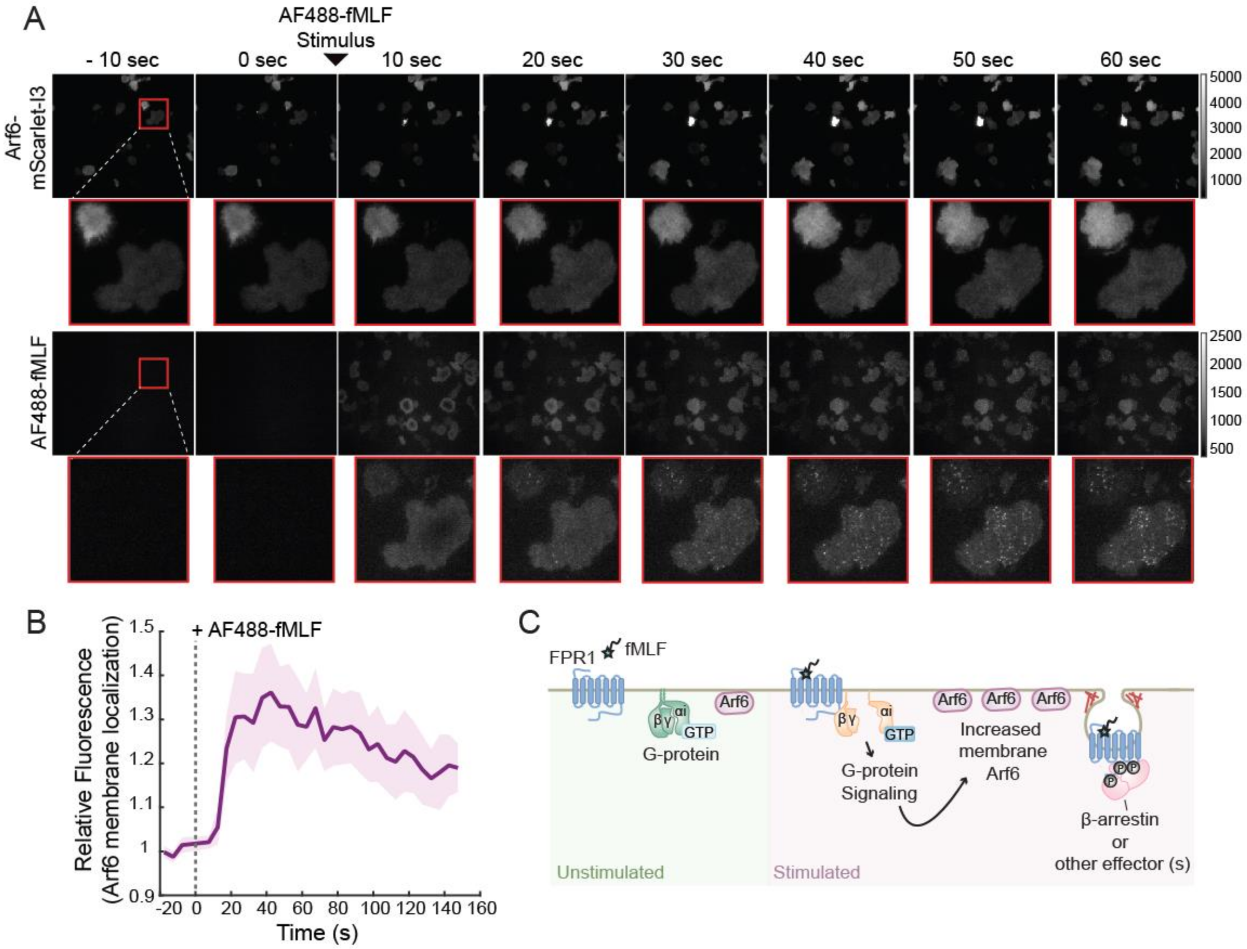

Minor:

1. Please specify which cell line was used for generating stable CRISPRi knockdowns of the different GRKs (GRK2, GRK3, GRK5, and GRK6), as this information is crucial for reproducibility.

All genetic editing is performed in HL-60 cells. We apologize that this was not clear. This information was given in the 'Cell maintenance and differentiation' subsection of the Methods section in the original manuscript. To make this more clear, we have added a reminder for this in the later section (Lines 686-687): "Stable expression of constructs, 3XHA-FPR1, dCas9, Cas9, CRISPRi and CRISPR sgRNAs, and mScarlet3i-Arf6 was achieved using 2nd generation lentiviral-mediated gene transfer into the HL-60 neutrophil-like cell line."

2. In the section "FPR1 uses both β-arrestin-dependent and β-arrestin-independent endocytic pathways," please correct the incomplete sentence ending with "…the knockouts in pooled cell liFigure S4)."

We apologize for this mistake. We have fixed this sentence (Lines 239-240) as “These results further confirmed the high efficiency of the knockouts in pooled cell lines (Supplementary Figure 5).”

3. In the section “Genome-wide CRISPR/Cas9 screens reveal regulators of basal FPR1 surface expression,” please add the appropriate figure or table reference to support the conclusion that “targeting mediators of neutrophil differentiation caused decreased basal surface FPR1 levels.”

We thank the Reviewer for the comment. The phrase “targeting mediators of neutrophil differentiation caused decreased basal surface FPR1 levels,” was a part of a topic sentence to introduce the shared hits between our FPR1 basal surface expression screen and the previously published differentiation screen (such as regulators of TOR). In addition, our screen captures other known neutrophil differentiation markers and identifies new ones as potentially important factors in this process. To clarify, we have added relevant figure and table calls to the sentence (Line 347-348) referred by the reviewer: “Consistent with this, we found that targeting mediators of neutrophil differentiation caused decreased basal surface FPR1 levels (Supplementary Figure 8 and Supplementary Table 4).”

4. Primary neutrophil isolation and purity assessment details are missing.

We thank the reviewer for asking about the methodological details of the neutrophil isolation. We agree that it is important to use pure primary cell populations to make conclusions on the effects of these inhibitors on human neutrophils. We used the EasySep™ Human Neutrophil Isolation Kit, a frequently used magnetic selection based negative selection method for human neutrophils. The efficiency of this kit has been tested by immunostaining of isolated neutrophil content (CD45+CD16+CD66b+) and found to achieve ~99% purity. We now added details on how the kit was used in our study in the Methods section (Lines 723-732) as follows:

“First, whole blood (~500 µl) mixed with 25 µl isolation cocktail (antibody mix that recognizes other cell types in blood than neutrophils) and 25 µl RapidSpheres (magnetic beads that will bind to labelled cells). After 5 minutes of incubation, this mix was topped by 4 mL PBS, containing 1 mM EDTA free of Ca++ and Mg++, and the tube was placed in an EasySep™ magnet (StemCell, catalog #18000). Unbound cell suspension was collected in a new tube and mixed with 25 µl RapidSpheres to capture the remaining labelled (non-neutrophil) cells. Similarly, unbound cell suspension is collected and subjected to magnetic separation two more times to achieve a highly pure neutrophil population. Immediately after isolation, cells were centrifuged at 250 g for 5 min and resuspended in pre-warmed complete RPMI medium to test effects of different inhibitors in FPR1 endocytosis assays as described below.”

As corroboration for the purity of the isolated neutrophils, we added a new Supplemental Figure (Supplementary Figure 3) showing the gating of a single, well-defined population and unimodal expression of FPR1, consistent with a population of pure neutrophils. This data corresponds to the same cells and gating as the data used in the main Figure 8C. In the main text (Lines 207-209), we added “To investigate the role of GRK proteins in primary human neutrophils, we isolated neutrophils, identifying a single population with uniform FPR1 staining by flow cytometry (Supplementary Figure 3).”

New Supplementary Figure 3: **Purity and uniform FPR1 staining of isolated human neutrophils.** (A) Gating strategy used for freshly isolated human neutrophils in flow cytometry experiments. (B) Uniform surface FPR1 staining of isolated neutrophils. Details of the protocol for neutrophil isolation are given in Methods.

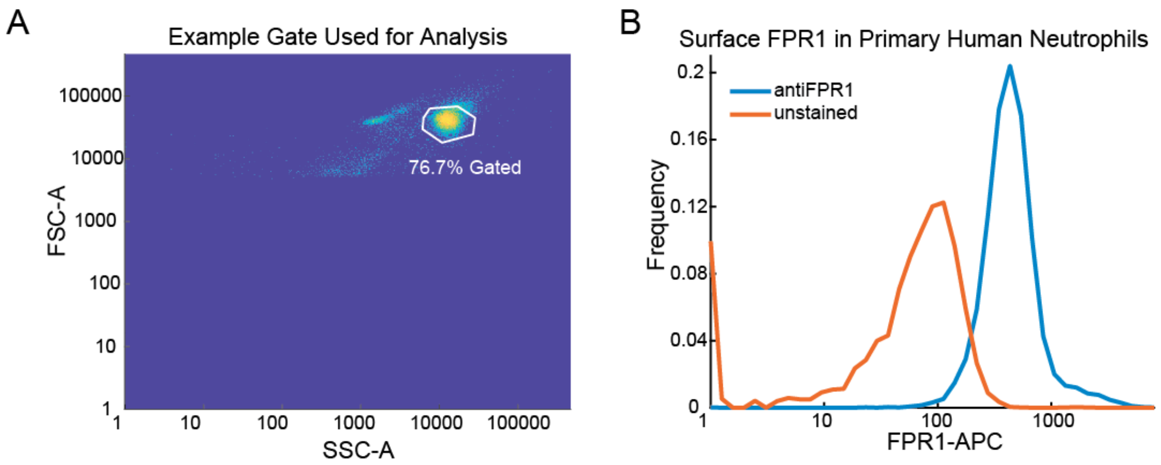

5. While the discussion appropriately acknowledges study limitations, it could better address potential off-target effects of the genetic perturbations and chemical inhibitors used, particularly given the reliance on chemical validation approaches.

We thank the reviewers for their concern about the off-target effects of gene editing strategies and chemical inhibitors used in our study. To better address the off-target effects of the genetic perturbations and chemical inhibitors, we added an additional reference, a new supplementary table with guide level scores, and more explanation in the main text of the revised manuscript.

Specifically,

- In the Results section, regarding the GRK2/3 inhibitor, we added a sentence (Lines 184-185) noting potential off-target activity, but also the specificity of this drug: "This compound has an EC50 at least 75-fold lower for these targets than for other known kinase targets," and the relevant reference[7].
- We also added a sentence in the Discussion (Lines 534-536) to note the intrinsic potential for off-target effects in genome-wide screens, but also emphasizing the strengths of our approach in this regard: "Though off-target effects are always a concern with screening approaches, our strategy used 10 independent sgRNAs per gene and more than 10,000 control guides, which provides a strong dataset for evaluating statistical confidence."
- Finally, to help readers assess possible off-target concerns for specific genes, we have added a new table (Supplementary Table 1), which includes the CLO, log-p-value, and Effect scores for every individual guide used in our screens. This will allow readers to identify the most active guides for a gene of interest and also assess whether the gene-

level score was driven by multiple distinct guides. We refer to this table in the main text where we first mention guide-level scores (Line 269): “We combine guide-level scores (Supplementary Table 1) to obtain gene-level scores (Supplementary Table 2), …”

We note that in our initial manuscript submission, we had already commented on off-target activities for SMIFH2 and NAV-2729. We had included references to the latest publications to our knowledge about these chemical inhibitors to make the reader aware of their potential off-target effects. For instance, in our submission, we mentioned in the Results section where we used an mDia1 inhibitor (Lines 454-456) that “at higher concentrations, SMIFH2 also inhibits myosin motors, and this dramatic internalization defect may be a result of multiple targets affected by the inhibitor.” Similarly, for the Arf6 inhibitor (Lines 479-480), NAV-2729, we “note that NAV-2729 can also inhibit the activity of ARF1, and to a lesser extent other regulators of the ARF GTPases.” We did not make any changes to these sentences.

References:

1. Li Y, Li Z, Zheng P, et al. Targeting formyl peptide receptor 1 reduces brain inflammation and neurodegeneration. *Science*. 2025;390(6774):eadq1177. doi:10.1126/science.adq1177
2. Subramanian BC, Moissoglu K, Parent CA. The LTB4–BLT1 axis regulates the polarized trafficking of chemoattractant GPCRs during neutrophil chemotaxis. *J Cell Sci*. 2018;131(18):jcs217422. doi:10.1242/jcs.217422
3. Beautrait A, Paradis JS, Zimmerman B, et al. A new inhibitor of the β-arrestin/AP2 endocytic complex reveals interplay between GPCR internalization and signalling. *Nat Commun*. 2017;8(1):15054. doi:10.1038/ncomms15054
4. Dahlgren C, Lind S, Mårtensson J, et al. G Protein Coupled Pattern Recognition Receptors Expressed in Neutrophils: Recognition, activation/modulation, signaling and receptor regulated functions. *Immunol Rev*. 2023;314(1):69-92. doi:10.1111/imr.13151
5. Morgens DW, Deans RM, Li A, Bassik MC. Systematic comparison of CRISPR/Cas9 and RNAi screens for essential genes. *Nat Biotechnol*. 2016;34(6):634-636. doi:10.1038/nbt.3567
6. Montagnac G, de Forges H, Smythe E, et al. Decoupling of Activation and Effector Binding Underlies ARF6 Priming of Fast Endocytic Recycling. *Curr Biol*. 2011;21(7):574-579. doi:10.1016/j.cub.2011.02.034
7. Okawa T, Aramaki Y, Yamamoto M, et al. Design, Synthesis, and Evaluation of the Highly Selective and Potent G-Protein-Coupled Receptor Kinase 2 (GRK2) Inhibitor for the Potential Treatment of Heart Failure. *J Med Chem*. 2017;60(16):6942-6990. doi:10.1021/acs.jmedchem.7b00443

There were no remaining issues raised by the reviewers. We have made small adjustments according to the Editorial Requests Table. Responses to all the editorial requests are included in the table.
